# Differential prevalence of PFAS, PCBs and pesticides in liver of hunted game

**DOI:** 10.1007/s11356-026-37805-w

**Published:** 2026-05-15

**Authors:** Alexandra Esther, Michelle Peter, Vera Ritz, Daniel Esther, Doreen Gabriel, Roman Trommler, Detlef Schenke, Klaus Polaczek, Kathrin Fisch, Christoph Müller

**Affiliations:** 1 Institute for Plant Protection in Horticulture and Urban Green, Julius Kühn Institute (JKI), Messeweg 11/12, 38104 Brunswick, Germany; 2 Department of Pharmacy, Center for Drug Research, Ludwig-Maximilians-Universität München, Butenandtstraße 5-13, 81377 Munich, Germany; 3 Department Pesticides Safety, German Federal Institute for Risk Assessment (BfR), Max-Dohrn-Straße 8-10, 10589 Berlin, Germany; 4 UFB Umwelt- und Forstbüro, Wilsdruffer Straße 25, 01737 Tharandt, Germany; 5 Institute for Crop and Soil Science, Julius Kühn Institute (JKI), Bundesallee 58, 38116 Brunswick, Germany; 6 Institute for Ecological Chemistry, Plant Analysis and Stored Product Protection, Julius Kühn Institute (JKI), Königin-Luise-Straße 19, 14195 Berlin, Germany; 7 Staatsbetrieb Sachsenforst, Kompetenzzentrum Wald und Forstwirtschaft, Bonnewitzer Straße 34, 01796 Pirna, Germany

**Keywords:** Wild boar (*Sus scrofa*), Roe deer (*Capreolus capreolus*), Liver residues, Liver consumption, Health risk, GC–MS/MS, LC–MS/MS

## Abstract

Free foraging game animals are constantly exposed to environmental pollutants and can therefore serve as bioindicators for these substances. However, they can also pose a health risk to humans if polluted meat of wild game animals enters the food chain. We analysed 219 potentially critical pollutants in 164 liver samples of hunted wild boar (*Sus scrofa*) and roe deer (*Capreolus capreolus*) from north-eastern Germany. Overall, 24 pollutants were detected and 16 of them were detected quantitatively. PCBs, PFAS and rodenticides were more frequent and present in far higher concentrations in wild boar than in roe deer. Only for rodenticides can it be assumed that there is no health risk for the game animals; otherwise, no statement can be made due to a lack of reference data. Risks for human consumers of liver arise from concentrations in wild boar liver exceeding the tolerable weekly intake for PFAS by up to 17 times and in individual cases the acute reference dose for brodifacoum by up to 485%. In contrast, the health risk of eating game meat is usually negligible, as chemical residues generally accumulate in the liver and less in muscle tissue. The significance of up to 12 co-occurring residues for animal and human health is still an open research issue.

**Graphical Abstract:**

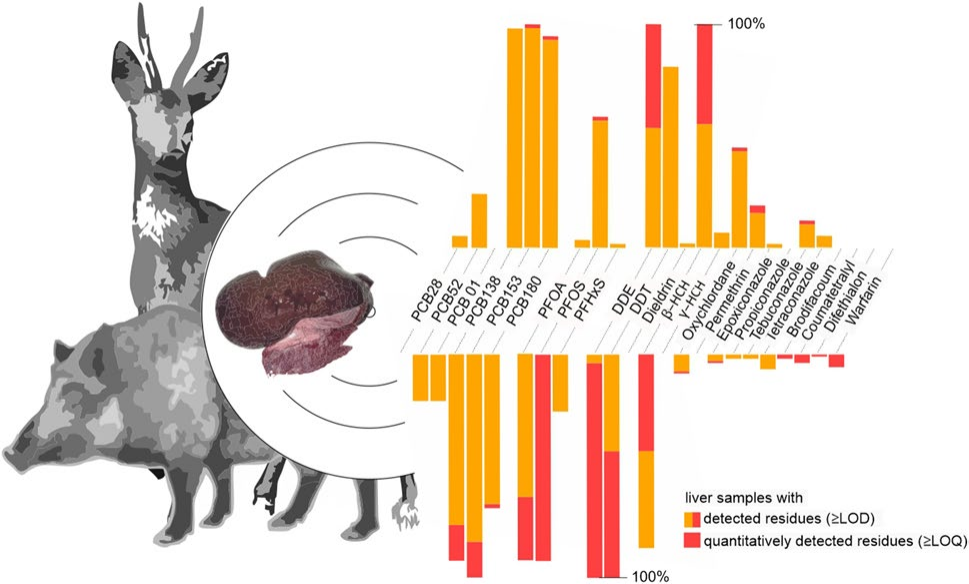

**Supplementary Information:**

The online version contains supplementary material available at 10.1007/s11356-026-37805-w.

## Introduction

For about two million years, the meat of hunted animals has supported the development of humankind, as it is nutritionally valuable due to its high protein and nutrient content (Lee and DeVore 2017; Ramanzin et al. 2010). Today, trade in meat from hunted game such as ungulates is common in many countries. Ungulates currently inhabit 90% of the European continent, with roe deer (*Capreolus capreolus*) and wild boar (*Sus scrofa*) occupying 74% and 64% of its land area (Linnell et al. 2020). Numbers of both species have increased over the past three decades with, for example, estimations of 15 million roe deer individuals in central Europe (Burbaitė and Csányi 2009; Linnell et al. 2020; Lovari et al. 2016). The damage they cause, e.g. by destruction and consumption of plants in agriculture and forestry, is associated with high annual economic costs exceeding 30 million Euros in Italy and France alone (Apollonio et al. 2017; Linnell et al. 2020). Hunting and meat marketing are part of management strategies for avoiding damage and conserving nature (Apollonio et al. 2017; Keuling and Leus 2019; Linnell et al. 2020). Regulations such as (EC) No. 853/2004 for European countries allow trading of wild game meat, which in turn supports the local economy of rural areas (Ramanzin et al. 2010).

Free-range and foraging game animals are constantly exposed to pollutants and can therefore serve as bioindicators of environmental pollution (Kaczyński et al. 2021; Ramanzin et al. 2010; Tomza-Marciniak et al. 2014). They can also pose a health risk to humans if polluted meat from the animals enters the food chain (Kaczyński et al. 2021; Petrović et al. 2022).

In recent years, one focus has been on pesticides and their possible health consequences. These include anticoagulant rodenticides (AR), which have been widely used for the control of rodents since the 1950 s (van den Brink et al. 2018). AR residues via primary exposure through direct consumption, secondary and/or tertiary exposure by consumption of polluted prey are frequent in many non-target species of mammalian carnivores like weasels, badgers and red foxes (Campbell et al. 2024; Keating et al. 2024) and avian predators like eagles, falcons and owls (Badry et al. 2021; Broughton et al. 2022; Moriceau et al. 2022), as well as scavengers like vultures (Moriceau et al. 2022; Oliva-Vidal et al. 2022). They were also found in hunted wild boars in Spain (Alabau et al. 2020). AR residues were more frequently detected in the liver than in the muscle and were associated with human population density and habitat anthropisation. Although the threshold of adverse effects on blood clotting was exceeded with liver residues of > 200 µg kg^−1^ for wild boars, the risk of acute poisoning to humans through consumption was assumed to be low (Alabau et al. 2020).

Persistent organic pollutants are hazardous to the environment and to human health and include organochlorine (OC) pesticides and polychlorinated biphenyls (PCBs) and are characterised by high chemical stability, low water solubility, semi-volatility and toxicity with a tendency towards accumulation in the environment (Lallas 2001). Particularly OC insecticides like dichlorodiphenyltrichloroethane (DDT), its metabolite DDE (dichlorodiphenyldichloroethylene), and compounds with the sum formula C_6_H_6_Cl_6_ like *gamma*-hexachlorocyclohexane (*gamma*-HCH also known as lindane) have been implicated in reproductive impairment, altered immune function and behavioural alteration (Vos et al. 2000). Strict regulations on these pollutants since the 1970 s led to substantial residue declines (Bustnes et al. 2007). However, they are still detectable in biota (Bustnes et al. 2007, Bustnes et al. 2022, Schnitzler et al. 2011, van den Steen et al. 2009). They were also found in muscle and liver samples of ungulates in Poland, with very low estimated chronic and acute risks to consumers (Kaczyński et al. 2021). Petrović et al. (2022) concluded for the lowland region of Serbia with intensive agriculture that the consumption of wild boar liver can be harmful to human health due to the sum of endrin aldehyde and ketone (Σ endrin), a stereoisomer of the OC insecticide dieldrin. For the risk assessment of pesticides for humans, acute reference doses (ARfD) and values for the acceptable daily intake (ADI) and adverse effect level (AEL) are available, which can be used to estimate intake via food without significant risk to consumers (e.g. EFSA et al. 2024).

PCBs originate from electrical equipment and numerous building materials such as flame retardants, plasticisers and paints (Montano et al. 2022). People are exposed to them through various exposure pathways, but mainly through the ingestion of contaminated food. A significant proportion of the PCBs detected in human serum, adipose tissue and breast milk are non-dioxin-like PCBs (NDL-PCBs), which primarily affect lipid metabolism and neurological functions (Montano et al. 2022). Given that six indicator NDL-PCBs (i.e. PCB 28, 52, 101, 138, 153 and 180) account for almost 50% of the total amount of NDL-PCBs in food, the AFSSA has established a tolerable daily intake (TDI) of 10 ng/kg bw/day for this group (AFFSA 2010). PCBs were found in wild boars and deer in neighbouring countries of Germany, in the Czech Republic (Maršálek et al. 2013) and in Poland (Petrović et al. 2022; Warenik-Bany et al. 2019).

In recent years per- and polyfluorinated alkyl substances (PFAS), a group of widely used man-made chemicals, have received increasing scientific and political attention because of their extreme persistency and mobility in the environment and organisms, potential toxicity with multiple adverse health effects (e.g. disruption of the endocrinological system and liver damage) and global presence also in remote regions (Brunn et al. 2023; González-Alvarez et al. 2024; Peritore et al. 2023). PFAS are used for a very wide variety of products, e.g. treatments for durable resistance to moisture and staining in clothing, furs, outdoor equipment, carpeting, fire-resistant surfactants in fire-fighting foams and chemical-resistant coatings in packaging. Wildlife exposure to PFAS takes place via contaminated water, soil, air and food. Within the group of PFAS, consisting of over 4700 man-made chemicals, perfluorooctanesulfonate (PFOS) and perfluorooctanoic acid (PFOA) are the most widely used and the most studied in terms of toxicokinetics and toxicodynamics. PFAS patterns are site specific and related to the local contamination source (Rupp et al. 2023). The major PFAS found in game studies are PFOS, PFOA and perfluorohexane sulfonate (PFHxS) (Brunn et al. 2023; Peritore et al. 2023). These PFAS are covered by the safety threshold set by the European Food Safety Authority (EFSA) in 2020, defining a group tolerable weekly intake (TWI) of PFOA, PFOS, PFHxS and perfluorononanoic acid (PFNA), of 4.4 ng kg^−1^ body weight per week for adults (Schrenk et al. 2020). The determination of a TWI is supposed to protect against increased PFAS concentrations. Elevated PFOS and PFOA levels, for example, in breast milk impair the immune response of infants to vaccines, or lead to an increase in serum cholesterol levels and a reduced birth weight (German Federal Institute for Risk Assessment 2021; Schrenk et al. 2020).

To investigate the potential risk of residues of pesticides (pollutants used as biocides or for plant protection), PFAS and PCBs in hunted game, we examined liver samples of roe deer and wild boar from Germany. We determined the concentrations of 219 chemical compound residues and assessed possible health consequences for the animals and potential human consumers. The study provides a valuable overview of current levels of individual pollutants with potential health risks in ecosystems and the food chain, as well as an important contribution to assessing the success of national and international agreements on the restriction of pollutants.

## Material and methods

### Sampling

The sampling of 58 roe deer and 106 wild boar livers was conducted between 2018 and 2021 in the German Federal States of Brandenburg and Saxony from sites with no known history of specific contamination. All sampling sites were dominated by forest and open land with 80–100%. The hunting of wild boar and roe deer as well as the removal of liver tissue during evisceration was carried out by the German Federal Institute for Risk Assessment and by hunters within the framework of hunting management all in compliance with the legal regulations and the necessary permits. All samples were stored either at −80 °C or −18 °C until analysis.

### Residues analysis

#### Rodenticides

The analysis was carried out in the laboratories of the Julius Kühn Institute in Berlin, Germany. The sample preparation and extraction of ARs in liver samples from hunted game follows the process diagram from Badry et al. (2021) (Table S1). In brief, after thawing, each sample was spiked with a surrogate mixture (acenocoumarol, diphacinone-*d*_4_, phenprocoumon, coumachlor). The liver was homogenised in methanol/water (2:1) and centrifuged. An aliquot of the supernatant was transferred by Supported Liquid Extraction (SLE) with an unbuffered diatomaceous earth (ChemElut) cartridge in dichloromethane. The eluate was concentrated to dryness, resolved in internal standard solution (chlorophacinone-*d*_4_ and warfarin-*d*_5_), filtrated and stored at −20 °C until measurement. The analytes were chromatographically separated by liquid chromatography gradient elution and the measurement was done with tandem mass spectrometry (LC–MS/MS) according to Badry et al. (2021) (Supporting Table S2). Identification and quantification of the rodenticides were performed after negative electrospray ionisation (ESI) with multiple reaction monitoring (MRM) and the comparison of the extended product ion spectra with reference spectra (EPI; acceptance limit > 80%) (Table S2). The concentrations of the ARs were calculated using the relative peak areas.

The analysis of the AR in the liver samples from wild boar and roe deer was carried out in two different analysis campaigns. The analytical procedure was verified by recovery experiments with untreated pig livers in the first (Table S2) and calf livers in the second campaign (Supporting Table S2), respectively, sourced from local grocery stores. Ongoing validation of the analytical procedure for the liver samples was also carried out via the recovery rates of the surrogates (Supporting Table S2). The matrix material used for quality control and the matrix calibration curves were without interferences. The matrix calibration curves were linear with *r*^2^ > 0.99 over the whole linear range from 0.01 to 20 pg/µl. All samples were measured in duplicate. The limit of quantification (LOQ) was determined according to SANTE/12682/2019. The LOQ is equal to the reporting limit and refers to the lowest calibration value with a signal-to-noise ratio > 6:1 and a relative standard deviation < 20% in the sequence. The concentrations of the ARs were not surrogate or recovery corrected.

#### Further prominent organic pollutants

The analysis was carried out by the Department of Pharmacy of the Ludwig-Maximilians-Universität München, Germany. The analysis of liver samples from roe deer (*Capreolus capreolus*) and wild boar (*Sus scrofa*) was conducted using two different miniaturised QuEChERS (quick, easy, cheap, effective, rugged and safe) approaches adapted to the physicochemical properties of the analytes. Gas chromatography-tandem mass spectrometry (GC–MS/MS) was used for the analysis of PCBs, organochlorine pesticides and further prominent pollutants according to the method of Schanzer et al. (2021). In total, the GC–MS/MS analysis covers 209 compounds, including six non-dioxin-like polychlorinated biphenyls (NDL-PCBs), 19 organochlorine (OC) insecticides, 51 other insecticides, 66 fungicides, 60 herbicides and 7 other compounds (e.g. nematicide, plant growth regulators) (Supporting Table S1 and S3).

Furthermore, the three most frequently detected PFAS (i.e. perfluorooctanoic acid (PFOA), perfluorooctane sulfonic acid (PFOS) and perfluorohexane sulfonic acid (PFHxS)) were analysed by LC–MS/MS. At the time of analysis, liver samples from 98 wild boars and 52 roe deers were available. The analysis was carried out using a combination of two well established assays, the GC–MS/MS approach by Schanzer et al. (2021) and the official method C-010.03 from the U.S. Food and Drug Administration (FDA) (FDA 2024). In short, 100.0 mg of liver was weighed into a 2.0-mL screw-cap tube containing two steel beads. Then, 100 µL of water and 200 µL of 1.5% formic acid in acetonitrile (*v/v*) were added. The tube was vortexed/homogenised for 15 min. Afterwards, 150.0 mg of anhydrous MgSO_4_:NaCl (4:1) were added and the tube was shaken immediately and then vortexed for 15 min. Afterwards, the tube was centrifuged for 5 min at 12,000*g* at room temperature and then stored at −20 °C for 2 h. An aliquot of 100 µL of the upper organic layer was transferred to a 0.5-mL microcentrifuge tube containing 21.0 mg of the dispersive solid phase mixture (dSPE; 15 mg anhydrous MgSO_4_, 5 mg PSA (primary secondary amine), 1 mg GCB (graphitised carbon black)). The tube was vortexed for 20 s and then centrifuged for 5 min at 12,000*g* at RT. At last, 70 µL internal standard solution (1 µg mL^−1^ sodium decanoic acid (SDS) in acetonitrile and water (95:5 (*v/v*))) was added to 70 µL supernatant, and the mixture was filtrated (Ø 13 mm, 0.2 µm, PTFE) before LC–MS/MS analysis (Supporting Table S4**)**.

Chromatographic parameters of organic pollutants (e.g. retention time, quantifier/qualifier transitions and limit of quantification (LOQ)) are listed in Table S1. All reported LOQs are in accordance with the previously published analytical method (Schanzer et al. 2021). The LOQ was defined as the lowest concentration with an overall bias and relative standard deviation below 20%. The limit of detection (LOD) was not determined according to SANTE/12682/2019. A compound was detected (> LOD) with a signal-to-noise ratio of 3:1, but the exact concentration was not determined. Detected residues were quantified (> LOQ) by interpolation against external calibration curves obtained using procedural standards (European Commission 2019). For quality assurance, the use of procedural standards allows compensation of matrix effects and avoidance of measuring errors. Procedural standards were prepared by spiking blank bovine liver samples from local grocery stores prior to extraction. The procedural standards were processed and measured by bracketing within the same batch as the analysed samples (20–30 samples per batch). If the response factors of the calibration standards at each concentration did differ by more than 30% and the deviation of the back-calculated concentrations did exceed 20% (European Commission 2019; Löbbert et al. 2021), the analysis, including sample preparation and measurement, was repeated.

### Data analysis and risk assessment

Determined concentrations refer to wet weight liver and were categorised as ‘not detected’ (< LOD) or ‘detected’ (≥ LOD), with the latter being further divided into ‘detected qualitatively’ (≥ LOD and < LOQ) and ‘detected quantitatively’ (≥ LOQ). Data was summarised as the arithmetic mean and median for which only samples with quantitatively detected residues were considered. Where it was necessary for comparisons with literature, values < LOQ were treated as zero for calculating the arithmetic mean and median.

To assess the health risk of liver consumption for humans, the values were calculated for a body weight of 70 kg (EFSA 2012), a weekly mean intake of 38 g liver for hunters, who are typically frequent liver consumers (Danieli et al. 2012), and a consumption of a portion of 200 g a year for other people (Rupp et al. 2023). To assess dietary exposure, the mean was calculated by considering values < LOQ as zero. The tolerable weekly intake (TWI) was used for risk assessment for PFAS and calculations based on Rupp et al. (2023). For risk assessment in terms of the NDL-PCBs, the tolerable daily intake (TDI) established by the French Food Safety Agency was used (AFFSA 2010). For conducting the human risk due to pesticides, acute reference doses (ARfD) and acceptable daily intake (ADI) values for active substances were used, published by the European Food Safety Authority (EFSA) and the European Chemical Agency (ECHA) (EFSA et al. 2024). Values of adverse effect level (AEL) were used when ARfD or ADI was not available. Critical concentrations are not available for multi-compound mixtures. For animal health risks due to anticoagulant rodenticides, estimations of the risk based on the established threshold of > 1000 µg kg^−1^ liver could be used (Newton et al. 1999; Thomas et al. 2011). Unfortunately, there are no thresholds for the other prominent pollutants in wildlife.

## Results

### Residues

**Fig. 1:**
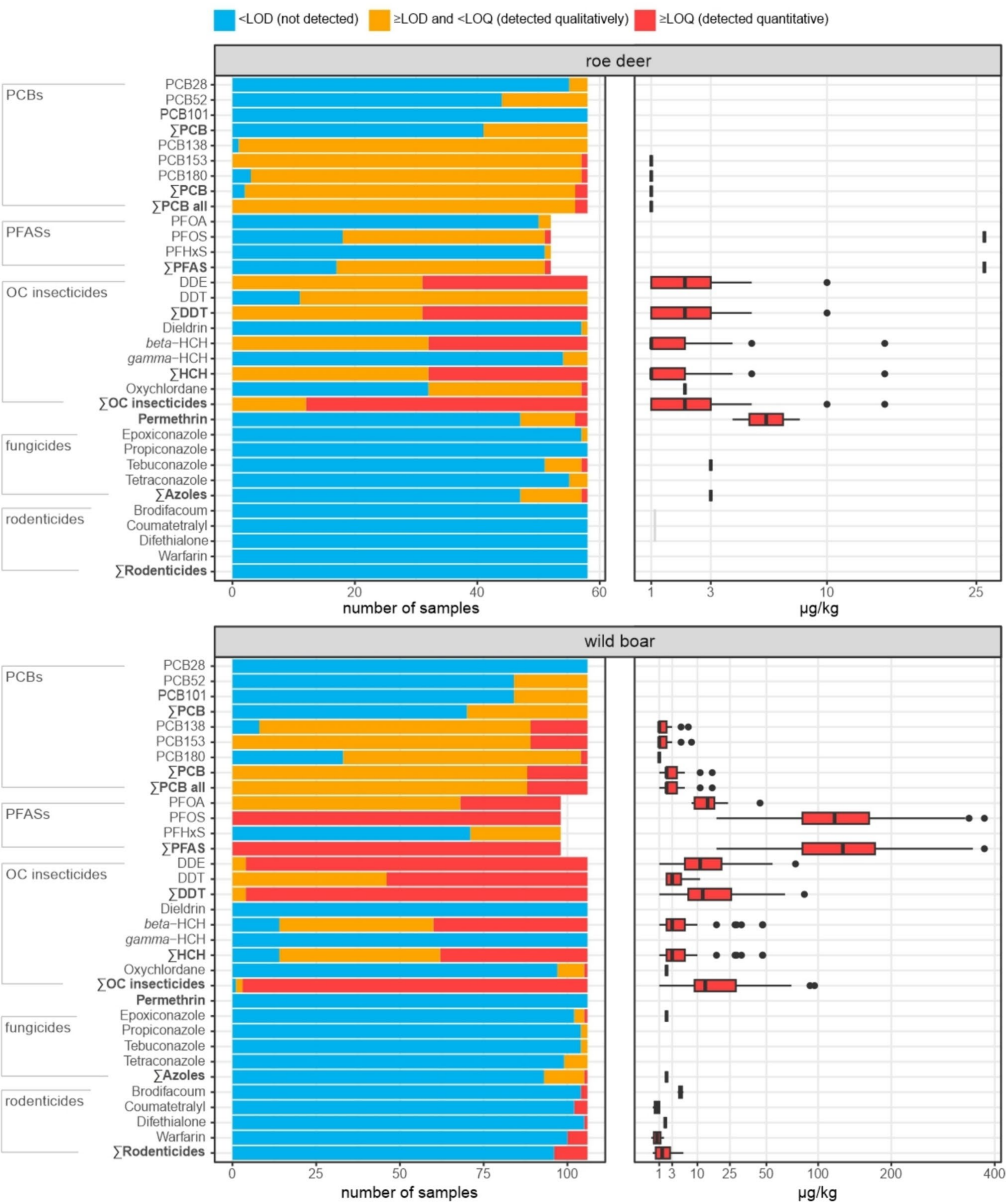
Frequency distribution of pollutants (left) and concentration ranges of pollutants detected quantitatively (right) in roe deer and wild boar samples (data available in Supporting Table S5). Boxplots on the right display median and lower and upper quartiles (minimum and maximum and points outside the interquartile range) of the detected concentration; *x*-axis is square root transformed

**Fig. 2:**
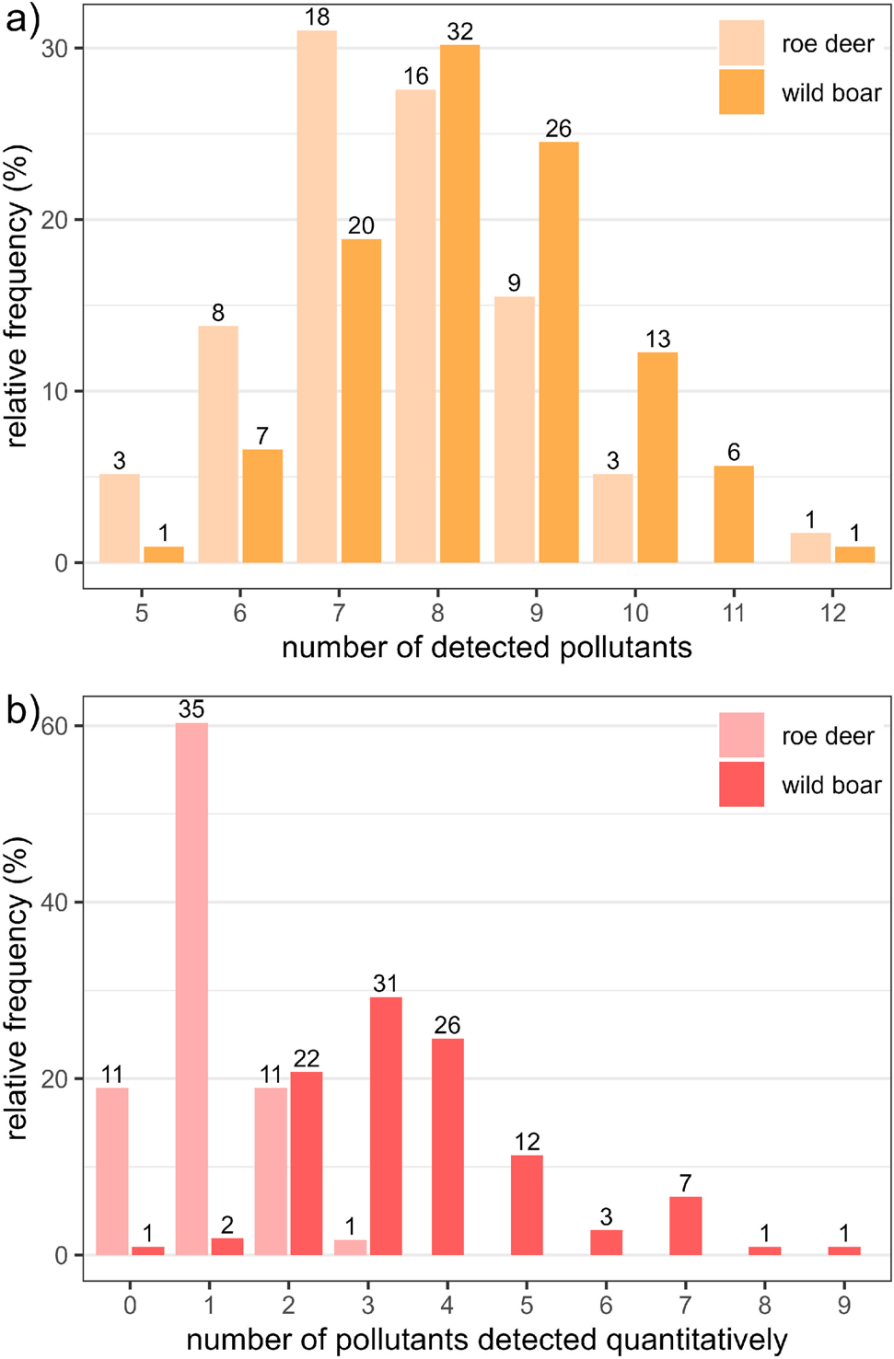
Relative frequency (%) of liver samples with (multiple) residues **a** detected (≥ LOD) and **b** detected quantitatively (≥ LOQ) in 58 roe deer and 106 wild boar. Numbers above the bars represent absolute numbers of samples

**Fig. 3:**
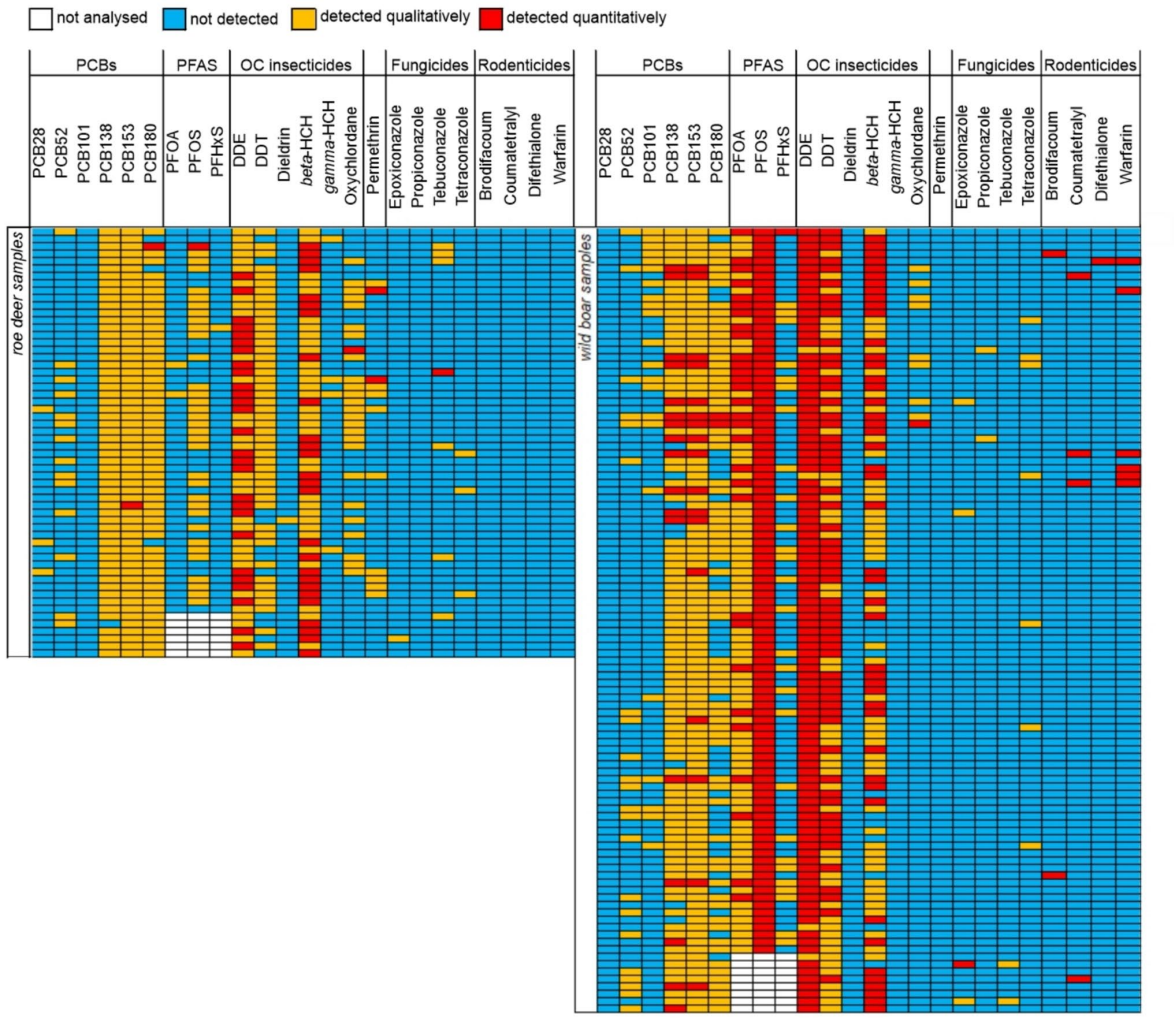
Detected pollutants in individual roe deer and wild boar samples

**Fig. 4:**
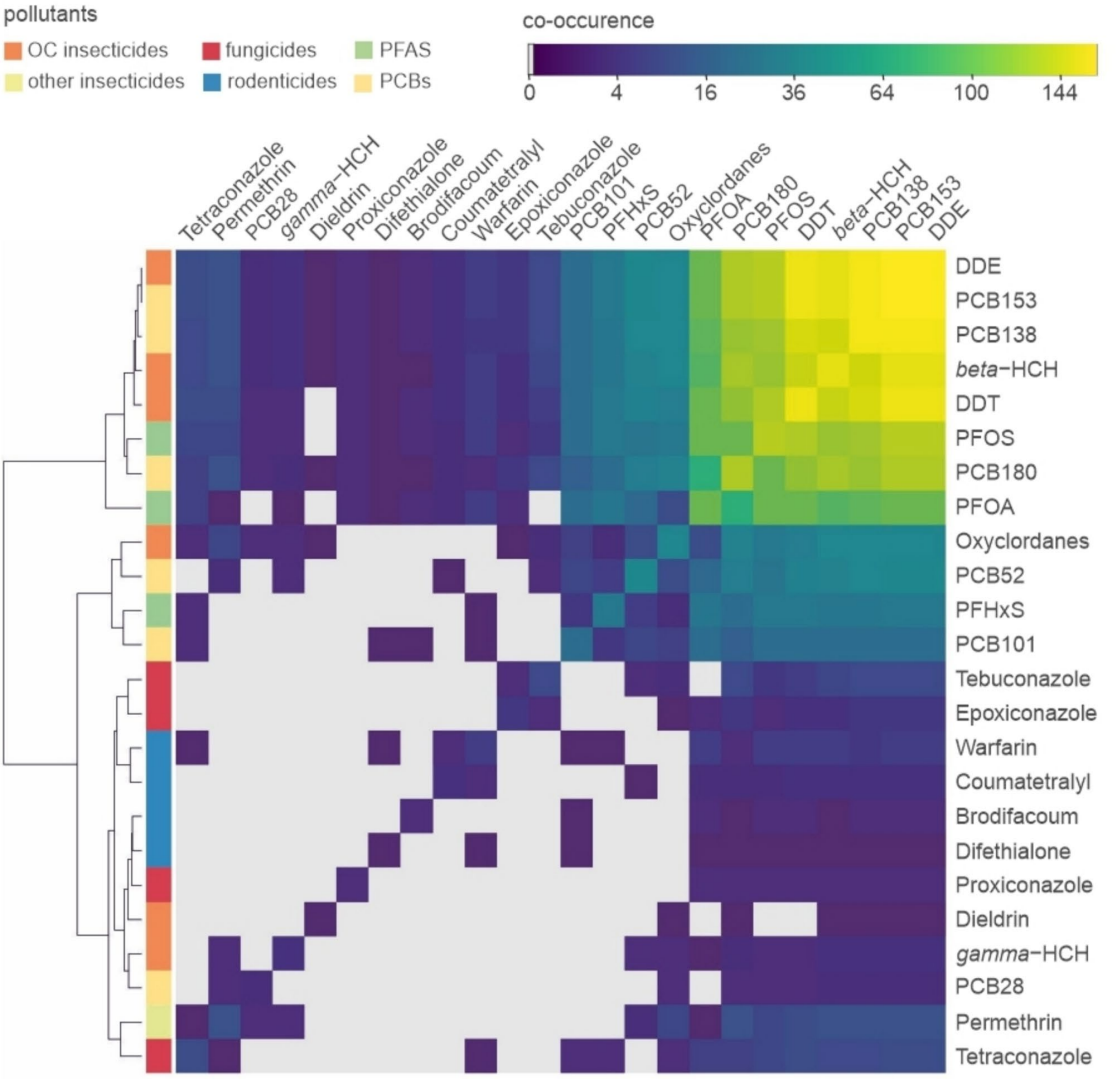
Co-occurrence of detected pollutants in individual samples. Dendrogram is based on Euclidean distances

In the liver samples, residues of 24 of the 219 investigated pollutants were detected and residues of 16 pollutants were quantified (Fig. 1, Supporting Tables S1 and S5). Each of the 164 samples contained 5–12 pollutants and up to 9 of these were quantified (Fig. 2). There are mixtures of PCBs, PFAS, insecticides (i.e. DDE, DDT, dieldrin, *beta*-HCH, *gamma*-HCH, oxychlordane and permethrin), fungicides (i.e. azoles) and rodenticides (Fig. 3). As expected, some substances frequently co-occur with others such as PCB138 with PCB153 and DDE with DDT and beta-HCH (Fig. 4).

### Residues in roe deer

OC insecticides were quantitatively detected in 79.3% of the 58 roe deer samples with up to 15 µg kg^−1^ (median 2 µg kg^−1^). The most frequently detected (46.2%) one was DDE (metabolite of DDT) with concentrations up to 10 µg kg^−1^ (median 2 µg kg^−1^), followed by beta-HCH with concentrations up to 15 µg kg^−1^ (median of 1 µg kg^−1^). DDT was detected but never at quantifiable concentrations (≥ LOQ of 2 µg kg^−1^). The azole fungicides tetraconazole, epoxiconazole and tebuconazole were detected in 11 samples and the latter was quantified in only one sample with 3 µg kg^−1^. Dieldrin and *gamma*-HCH were detected but not quantifiable (< LOQ) in one and four samples, respectively. The insecticide permethrin was detected in six samples and quantified in two samples with a maximum of 8 µg kg^−1^ (median 6 µg kg^−1^).

NDL-PCBs were detected in all 58 roe deer samples (Fig. 1). The shorter half-life PCB28, PCB52 and PCB101 and the longer half-life PCB138 were detected in 5.2–98.3% of the roe deer samples but never quantified (< LOQ). The more persistent PCB153 and PCB180 were detected in 100% and 94.8% of the samples and were quantified in only one sample at 1 µg kg^−1^ each. PCB28 was detected in 5.2% of the roe deer samples.

PFAS were detected in 67.3% of the 52 investigated roe deer samples, PFOS in 65.4%, PFOA in 3.8% and PFHxS in 1.9%. PFAS were quantified in only one roe deer sample (26 µg kg^−1^ PFOS). Rodenticides were not detectable in the 58 roe deer samples (Fig. 1).

Overall, concentrations of multiple pollutants were found in 81% of the roe deer samples with a mean concentration of 3.4 µg kg^−1^ and a maximum concentration of 42 µg kg^−1^ (Table 1). At most, ten pollutants were detected in one sample and three pollutants were quantifiably detected (Fig. 2).

**Table 1:** Amounts of pollutants which were quantified (≥ LOQ) in roe deer and/or wild boar

Residues	Roe deer				Wild boar			
	Samples	Mean	Median	Max	Samples	Mean	Median	Max
	[%]	[µg kg^−1^]	[µg kg^−1^]	[µg kg^−1^]	[%]	[µg kg^−1^]	[µg kg^−1^]	[µg kg^−1^]
∑PCBs	3.4	1.0	1.0	1.0	17.0	3.7	2.0	16.0
PCB138					16.0	1.94	1.0	7*.0*
PCB153	1.7	1	1	1	16.0	1.94	1.0	8.0
PCB180	1.7	1	1	1	1.9	1.0	1.0	1.0
∑PFAS	1.9	26.0	26.0	26.0	100	139.1	130.0	377.0
PFOA					30.6	14.8	14.0	45.0
PFOS	1.9	26.0	26.0	26.0	100	133.6	119.5	377.0
∑OC insecticides	79.3	2.5	2.0	15.0	97.2	20.8	13.0	96.0
DDE	46.6	2.3	2.0	10.0	96.2	15.4	11.0	76.0
DDT					56.6	4.14	1.0	11.0
*beta*-HCH	44.8	2.1	1.0	15.0	43.4	7.4	3.0	47.0
Oxychlordane	1.7	2.0	2.0	2.0	0.9	2.0	2.0	2.0
***other insecticides***	3.4	6.0	6.0	8.0				
Permethrin	3.4	6.0	6.0	8.0				
∑Fungicides	1.7	3.0	3.0	3.0	0.9	2.0	2.0	2.0
Epoxiconazole					0.9	2.0	2.0	2.0
Tebuconazole	1.7	3.0	3.0	3.0				
∑Rodenticides					9.4	2.0	1.4	5.6
Brodifacoum					1.9	4.9	4.9	5.6
Coumatetralyl					3.8	0.8	0.8	1.1
Difethialone					0.9	1.8	1.8	1.8
Warfarin					5.7	0.9	0.8	1.6
∑Multiples	81.0	3.38	2.0	42.0	99.1	151.0	140.8	413.0

### Residues in wild boar

OC insecticides were detected in all 106 wild boar liver samples and fungicides were detected in 12.2% of the wild boar samples (Table 1). They were quantified in 97.2% of the samples up to 96 µg kg^−1^ (median 13 µg kg^−1^). DDE was quantified in concentrations of up to 76 µg kg^−1^ (96.0% of the samples; median 11 µg kg^−1^), DDT in concentrations of up to 11 µg kg^−1^ (56.6% of the samples; median 3 µg kg^−1^) and *beta*-HCH with a concentration of up to 47 µg kg^−1^ (median 13 µg kg^−1^).

The azole fungicides propiconazole, tetraconazole, tebuconazole and epoxiconazole were detected; however, only epoxiconazole was quantified in one sample at 2 µg kg^−1^. Permethrin, dieldrin and *gamma*-HCH were not detected in wild boar samples.

NDL-PCBs were detected in all 106 wild boar samples. In 17.0% of the samples, PCBs were quantified with a maximum of 16 µg kg^−1^ (median 2 µg kg^−1^). The longer half-life PCB153 and PCB138 were detected in 92.2% and 100%, respectively, and were quantified in 17 samples (maximum 7 µg kg^−1^ and 8 µg kg^−1^, median for both 1 µg kg^−1^). The shorter half-life PCB52 and the longer half-life PCB180 were present in 20.8% and 68.9% of the samples, respectively. PCB180 was quantified in two samples with a maximum of 1 µg kg^−1^ PCB101 which was only detected in wild boar samples (20.8%, value < LOQ).

PFAS were detected in all 98 investigated wild boar samples (Fig. 1), especially PFOS. PFOS was quantified in all samples with concentrations of up to 377 µg kg^−1^ (median 119.5 µg kg^−1^). PFOA was detected in all samples and quantified in 30.6% of samples with a maximum concentration of 45 µg kg^−1^ (median 14 µg kg^−1^). PFHxS was detected in 27.6% of the wild boar samples but was not quantified (< LOQ of 8 µg kg^−1^).

Rodenticides were detected in ten of the 106 wild boar samples. Warfarin was quantified in 5.7%, coumatetralyl in 3.8%, brodifacoum in 1.9% and difethialone in 0.9% of the samples. The maximum concentrations were 1.6 µg kg^−1^ (median 0.9 µg kg^−1^) for warfarin, 1.1 µg kg^−1^ (median 0.8 µg kg^−1^) for coumatetralyl and 5.6 µg kg^−1^ (median 4.9 µg kg^−1^) for brodifacoum. Difethialone was quantified at 1.8 µg kg^−1^. Three samples contained two rodenticides; two samples contained warfarin and coumatetralyl of 2.3 µg kg^−1^ and 2.7 µg kg^−1^, and one contained warfarin and difethialone of 2.1 µg kg^−1^.

Overall, multiple pollutants were found in 99.1% of the wild boar samples with a mean concentration of 151 µg kg^−1^ and a maximum concentration of 413 µg kg^−1^ (Table 3). At most, 12 pollutants were detected in one sample and nine pollutants were detected qualitatively (Fig. 2).

### Risk for human consumers of liver

The mean concentration of PFAS in wild boar livers exceeds the tolerable weekly intake (TWI) of 4.4 µg kg^−1^ body weight for hunters by more than 17 times (Table 2). Even a one-time intake of 200 g liver per year exceeds the threshold by more than 1.7 times. The threshold is not reached if the liver of roe deer is consumed, as only very few PFAS were present.

**Table 2:** Risk assessment for human dietary exposure to PFAS and PCBs through liver consumption. Individual concentrations below LOQ have been set to zero for the calculations

	Roe deer	Wild boar
Mean of ∑PFAS-3^*^ µg kg^−1^	0.5	139.1
(a) Weekly mean intake in µg kg^−1^ body weight via consumption of 0.038 kg liver **/****	0.27 × 10^−3^ ≙ 0.06 × TWI	75.51 × 10^−3^ ≙ **17.16 × TWI**
(b) Annual intake in µg kg^−1^ body weight via consumption of 200 g liver expressed as weekly intake ***/****	0.027 × 10^−3^ ≙ 0.01 × TWI	7.64 × 10^−3^ ≙ **1.73 × TWI**
∑PCB µg kg^−1^	0.034	0.63
(a) Daily mean intake in µg kg^−1^ body weight via consumption of 0.038 kg liver per week**/*****	2.6 × 10^−6^ ≙ 0.0003 × TDI	49 × 10^−6^ ≙ 0.005 × TDI

^*^∑PFAS-3 is sum of PFHxS, PFOS, and PFOA measured in present study; the amount of PFNA was not determined in this study

^**^Normalised to a human body weight of 70 kg (EFSA 2012).and calculated with the mean amount of wild boar liver (38 g week^−1^) consumed by the Italian hunter population (Danieli et al. 2012)

^***^Normalised to a human body weight of 70 kg (EFSA 2012) using the consumption of a portion of 200 g (Rupp et al. 2023)

^****^TWI (tolerable weekly intake) = 4.4 × 10^−3^ µg kg^−1^ body weight for sum concentrations of PFHxS, PFOS, PFOA and perfluorononanoic acid (PFNA) and (ΣPFAS-4) (EFSA et. al, 2024)

^*****^TDI (tolerable daily intake) = 0.01 µg kg^−1^ body weight per day for the group of non-dioxin like PCBs (i.e. PCB-28, 52, 101, 138, 153 und 180) (AFSSA 2010)

The mean concentration of NDL-PCBs in wild boar and in roe deer livers was ≤ 0.005 times lower than the tolerable daily intake (Table 2). The concentrations of most pesticides were below 3% of ARfD and ADI. Concentrations of rodenticides in wild boar were the exception (Table 3). The concentrations of difethialone and coumatetralyl corresponded to 30.3% and 10.1% of the ARfD, respectively. The concentrations of brodifacoum corresponded to 484.8% of the ARfD.

**Table 3:** Acute and chronic risk assessment for pesticides. Individual concentrations below LOQ have been set to zero for the calculations

			Roe deer				Wild boar			
Residues	ARfD*	ADI**	Max	ARfD	Mean	ADI	Max	ARfD	Mean	ADI
	[μg kg b.w.^-1^]	[μg kg b.w.^-1^]	[μg kg^-1^]	[%]***	[μg kg^-1^]	[%]****	[μg kg^-1^]	[%]***	[μg kg^-1^]	[%]****
*OC insecticides*										
DDTs (∑DDE,DDT)^1^	10	10	10	0.3	1.09	< 0.1	85	2.4	16.96	0.1
*beta*-HCH^2^	60	0.5	15	< 0.1	0.95	0.1	47	0.2	3.21	0.3
Oxychlordane^3^	0.5	0.5	2	1.1	0.03	< 0.1	2	1.14	0.02	0.1
*Other insecticides*										
Permethrin^4^	10	10	8	0.2	0.21	< 0.1				
*Fungicides*										
Epoxiconazole^4^	23	8					2	<0.1	0.02	<0.1
Tebuconazole^4^	30	30	3	< 0.1	0.05	< 0.1				
*Rodenticides*										
Brodifacoum^5^	0.0033	0.0033					5.6	484.8	0.09	1.5
Coumatetralyl^6^	0.031	0.017					1.1	10.1	0.03	0.1
Difethialone^7^	0.017	0.007					1.8	30.3	0.02	0.2
Warfarin^8^	67	2					1.6	< 0.1	0.05	<0.1

*Acute references doses for active (ARfD) substance; also AELacut oder AELshort-term

**Acceptable daily intake value, also AELlong-term or AELsubchronic

***Calculated with consumption of one portion of 200 g a year (Rupp et al. 2023) and normalized to a human body weight of 70 kg (EFSA 2012)

**** calculated with mean amount of wild boar liver (38 g week^-1^) consumed by the Italian hunter population (Danieli et al. 2012) and normalized to a human body weight of 70 kg (EFSA 2012)

^1^https://apps.who.int/pesticide-residues-jmpr-database/pesticide?name=DDT

^2^https://mobil.bfr.bund.de/cm/343/bfr_sieht_keine_gesundheitsgefahr_durch_ueberhoehte_hch_gehalte in_fischen_aus_mulde_und_elbe.pdf und 10.17590/20240628-134359-0

^3^https://www.efsa.europa.eu/sites/default/files/2021-04/6491.pdf

^4^EFSA et al. (2024)

^5^https://echa.europa.eu/documents/10162/fa3f5493-6089-bbf3-ec81-84b79b56f259

^6^https://echa.europa.eu/documents/10162/8222fd05-0570-00ff-e7e7-73d9bcd33433

^7^https://echa.europa.eu/documents/10162/d92237fb-4a5a-b1ef-c0e7-c8f009a12a9d

^8^https://echa.europa.eu/documents/10162/141566ca-34c6-6e09-a9b9-edc451cb1eec

## Discussion

### Residues

There is no monitoring programme for chemical residues in hunted game animals in Europe. Individual studies found residues of rodenticides (Alabau et al. 2020; Coeurdassier et al. 2014; Drašković et al. 2023; Keating et al. 2024), OC pesticides (Kaczyński et al. 2021; Maršálek et al. 2013; Petrović et al. 2022), PCBs (Maršálek et al. 2013; Petrović et al. 2022; Stadion et al. 2024; Warenik-Bany et al. 2019, 2016) and PFAS (Guckert et al. 2023; Kowalczyk et al. 2018; Rupp et al. 2023). Our study investigation of 219 of these pollutants showed that residues of 24 can be detected, 16 of them at quantifiable levels.

As is the case for all pollutants analysed in the present study, the widespread use of anticoagulant rodenticides leads to an exposure risk for non-target species due to their bioaccumulative and persistent properties. Studies point to the risk of residues but analysis on game animals is seldom (Alabau et al. 2020; Drašković et al. 2023). Game animals can be affected by indirect and direct consumption of rodenticides, as is the case for the omnivorous species wild boar (Alabau et al. 2020). This is supported by the recent study, wherein 9.4% of the analysed liver samples from wild boar had anticoagulant residues. Overall the concentrations with a maximum of 5.6 µg kg^−1^ were lower than reported for rural areas in Spain with 8.7 µg kg^−1^ (Alabau et al. 2020). In urban and suburban areas, concentrations up to 678 µg kg^−1^ were found, without correlations of liver concentrations with sex and age (Alabau et al. 2020). Highest concentrations were found for the potentially bioaccumulating, persistent and toxic second-generation anticoagulant brodifacoum. This may also be due to the fact that this is one of the most commonly used anticoagulant rodenticides (Geduhn et al. 2015).

Persistent organic pollutants (POPs) include OC pesticides and PCBs, which are known for their resistance to biological and chemical degradation in relation to dietary exposure and toxicity. There is also a long-standing environmental concern about them in hunted wild ungulates used as meat sources (Capcarova et al. 2019; Kaczyński et al. 2021; Petrović et al. 2022). In accordance with the current study, OC insecticides like DDT and *beta*-HCH are commonly found (Table 1) (González-Gómez et al. 2021; Petrović et al. 2022). The mean concentrations of 15.4 µg kg^−1^ for DDE and 4.14 µg kg^−1^ for DDT found in wild boar livers are lower than in neighbouring country Poland with a mean value of > 67 µg kg^−1^ (Tomza-Marciniak et al. 2014). Spatial and temporal variations are known and effects of age and sex are possible (Capcarova et al. 2019; Maršálek et al. 2013; Naso et al. 2004). In general, concentrations in muscle meat appear to be lower than in liver (Kaczyński et al. 2021; Maršálek et al. 2013; Naso et al. 2004). Due to their high lipophilicity, OC pesticides and POPs tend to bioaccumulate more in fatty tissues, like liver, than in muscle (Amutova et al. 2021).

In addition to OC insecticides, other insecticides were quantified in the current study in one to two samples, i.e. the insecticide permethrin and the fungicides epoxiconazole and tebuconazole. Only tebuconazole is currently still authorised as a plant protection product, whereas the expiry date of epoxiconazole was 2021 (authorisation ended in 2019), after the data collection for the current study. Findings of such pollutants seem to vary greatly depending on application. For example, permethrin was not found in wild boar in the current study in accordance with a study from the neighbouring country Poland (Kaczyński et al. 2021) and in contrast to residues in 85% of the samples in Spain (González-Gómez et al. 2021). In Poland, tebuconazole was the most common fungicide with about 24% of the wild boar as well as of the roe deer samples, but in the current study, it was detected only in 12% of roe deer and in 3% of wild boar liver samples.

Another kind of POPs we found were NDL-PCBs. They were detected in all liver samples of the hunted games which is in accordance with other studies (Warenik-Bany et al. 2016, 2019). In contrast to low-chlorinated PCBs with a shorter half-life (PCB28, PCB52 and PCB101), high-chlorinated PCBs with a longer half-life (PCB138, PCB153 and PCB180) were present in almost all samples and detected quantitatively (Fig. 1, Table 1). Mean NDL-PCB concentrations for wild boar of 3.7 µg kg^−1^ were similar to those determined in a study from Serbia with 5.5 µg kg^−1^ (Petrović et al. 2022). The maximum measured quantity in the current study (16 µg kg^−1^) was, however, higher than the maximum measured in Germany’s first diet study with 0.184 µg kg^−1^ for wild boar meat (Stadion et al. 2024). Residue variation can be explained by the differences in sample preparation. Manufacturing processes such as defrosting, cooking and frying could lead to a reduction of non-dioxin-like PCBs (JECFA (2016)). Further, liver tissue can contain higher PCB concentrations than commonly eaten muscle meat. Warenik-Bany et al. (2016) found a liver/muscle ratio of about 2.7 for roe deer and of 1.1 for wild boars. Animals’ age does not appear to influence PCB concentrations, though sex might (Maršálek et al. 2013; Warenik-Bany et al. 2016). Pollutant levels, types and their relative abundance in deer and boar tissue could reflect their surrounding environment and concentrations in agricultural areas may be lower than in industrial areas (Warenik-Bany et al. 2019).

Due to the persistence and long-range transport of PFAS in the atmosphere, they are widely distributed and have already been measured in various environmental compartments such as in water, soil and wildlife species (Brunn et al. 2023; Death et al. 2021; Giesy and Kannan 2001; Panieri et al. 2022). In accordance with other studies, we found PFAS in the liver samples of hunted games (Death et al. 2021; Kowalczyk et al. 2018; Stahl et al. 2012). In our study, residues were found in wild boar but, with one exception (26 µg kg^−1^ for PFOS), not in roe deer. PFOS concentrations reached up to 377 µg kg^−1^ (mean 133 µg kg^−1^) and were more frequent and much higher than PFOA concentrations of up to 45 µg kg^−1^ (mean 14.8 µg kg^−1^). The marked differences between PFOS and PFOA concentrations have been documented. Kowalcyk et al. (2018) found a mean PFOS concentration of 179 µg kg^−1^ in the livers of wild boars reaching up to 1084 µg kg^−1^, whereas PFOA concentrations were considerably lower with a mean of 8.8 µg kg^−1^ and a the maximum of 114 µg kg^−1^ (Kowalcyk et al. 2018).

The values we found for PFOS in wild boar liver were within the known ranges for Germany (Rupp et al. 2023). Rupp et al. (2023) found PFOS concentrations that were multiple times higher in wild boar liver on known contaminated farmland than in areas with non-specific background contamination (426 µg kg^−1^ vs 82 µg kg^−1^), which reflected contamination of the local soil. Regional differences are likely, as higher concentrations can be expected in areas with high population density and in known PFAS hotspots with severe contamination (Kowalczyk et al. 2018). In addition, further environmental samples from the relevant areas would have to be examined in order to draw conclusions about correlations between sources of pollutants and residues in animal livers; however, no further data on environmental pollutants from the relevant areas are available.

The absence of data for individual animals and earlier sampling periods restricts further analyses in the present study. Falk et al. (2012) reported declining PFOS concentrations more than PFOA concentrations in roe deer liver between 2000 and 2010, while Kowalcyk et al. (2018) described age-related, but not sex-related, differences in PFOS concentrations in wild boar.

### Risk for animals

There is a lack of studies reporting on the relationship between liver residues in animals and their health (Death et al. 2021). The focus is commonly on consumed concentrations without looking for liver accumulation and not on long-term exposure by doses in concentrations found in the environment (Peritore et al. 2023). One livestock study with juvenile chickens (*Gallus gallus*) found mean concentrations of 4550 µg kg^−1^ for PFOS and 950 µg kg^−1^ for PFOA in the liver after three weeks of high-dose exposure, but no adverse effects on body weight, organ indexes, blood clinical parameters or organ histopathology (Yeung et al. 2009). Concentrations found in the current study, in mean 26.0 µg kg^−1^ for roe deer liver and 139.1 µg kg^−1^ for wild boar liver, were significantly lower based on permanent exposure to environmental concentrations. Chickens’ short-term exposure to high doses of PFAS is not likely to have the same health effects. In general, although concern has been raised over the toxic effects of environmental pollution, it is presently unclear to what extent liver residues indicate a risk for wild animals such as game animals. One exception which has been established within the scientific community is anticoagulant rodenticides. A threshold of 1000 µg kg^−1^ in animal liver is commonly used for showing possible consequences for animal health (Newton et al. 1999; Thomas et al. 2011). Maximum concentrations of 5.6 µg kg^−1^ in the current study are clearly below the threshold. However, it cannot be ruled out that the observed contaminants negatively influence animal health despite their concentrations being below individual thresholds. When combined, dose-additive effects can occur, where each contaminant contributes more or less to an overall effect, even when individual doses would not be harmful (Kortenkamp 2007). However, the effect of pollutant mixtures can be also synergistic or antagonistic, and substances classified as low toxicity can significantly increase overall toxicity (Göbölös et al. 2024). For instance, it has been demonstrated that non-toxic or low-toxic substances, such as boscalid and terbuthylazine, have the capacity to significantly reduce the LD50 values of pesticides, thereby increasing their toxicity (Tsvetkov et al. 2017). The simultaneous detection of 12 chemical substances with largely unknown interactions has the potential to have harmful effects on wildlife species such as wild boar and red deer.

### Risk for human consumers of liver

High residue levels of PFAS and, even if sporadic, of rodenticides support the general recommendations of the German Federal Institute for Risk Assessment not to consume wild boar liver (German Federal Institute for Risk Assessment 2024). The extent to which this applies only to some regions cannot be confirmed by the study due to the sample restriction, but nationwide monitoring with spatially and temporally explicit analysis could provide sufficient data.

Nevertheless, regardless of the assumptions of whether 200 g of liver is eaten once a year or a weekly average of 38 g is consumed (Danieli et al. 2012), the TWI of 4.4 µg kg^−1^ body weight for PFAS is exceeded (Schrenk et al. 2020) (Table 2). The values are in the range reported from German areas, although the concentrations are comparable with those determined in the area with a historic case of contamination (5 times and 18 times TWI) and lower than the residues found in the industrial area (5 and 33 times TWI) (Rupp et al. 2023). However, they are higher than those in areas with background contamination of 1.3 and 4 times TWI, respectively.

The TDI of 10 ng/kg body weight/day for NDL-PCBs is not exceeded for the consumption of wild boar and roe deer liver (Table 2). This type of assessment indicates that there is no risk to consumers of wild boar and roe deer liver. This is in contrast to the analysis of Petrović et al. (2022) and Warenik-Bany et al. (2016), who identified a risk.

The risk for human health posed by pesticides seems to be low because residues are found in much lower concentrations than ARfD and ADI (Table 3), which also corresponds with results from Poland (Kaczyński et al. 2021). The exception is individual wild boar cases, because brodifacoum residues exceeded the ARfD, which indicates an acute health risk if a portion of 200 g of wild boar liver is consumed (Table 3).

In general, the influence of manufacturing processes such as defrosting, cooking and frying on compound concentrations in the liver has not yet been clarified. Different cooking methods could either reduce or increase the levels of chemical contaminants in food (Domingo, 2011). The scientific literature is inconsistent regarding the impact of cooking and food processing on exposure (Domingo, 2011; Schrenk et al. 2020; Stadion et al. 2024).

Various consequences of exposure to single or groups of compounds for human health are known. Anticoagulant rodenticides can inhibit the vitamin K cycle in the liver leading to inhibition of blood coagulation and haemorrhage. Sub-lethal, asymptomatic effects based on coagulant-independent cells and tissues are also possible (Popov Aleksandrov et al. 2024). Having been mostly derived from animal trials with higher doses than those to which humans are exposed, possible health consequences of PFAS include liver toxicity, immune toxicity and increased risk for cardiovascular diseases (Death et al. 2021, Schrenk et al. 2020). In contrast, consequences of multiple residues are still ultimately unknown because of the complexity of the topic (Caldas 2023). Defining a cumulative assessment group depending on a sound and high-quality toxicological database is seen as a main challenge (Caldas 2023). EFSA et al. (2024) determined that the presence of multiple residues within a single sample remains permissible, as long as each individual residue level does not exceed the individual maximum residue level for each active substance, which is what the current study shows for PFAS and rodenticides.

### Game liver as a bioindicator

Wild animals are often suggested as bioindicators of environmental pollution for single or groups of pollutants (Guckert et al. 2023; Kowalczyk et al. 2018; Rupp et al. 2023). Wild boars can be good bioindicators and are especially quantitatively suitable for determining the presence of PFAS in the environment (Kowalczyk et al. 2018). Rupp et al. (2023) conclude from PFAS profiling that wild boars seem to represent the environmental contamination in soil. Wild boars are known as omnivores and burrowers; they may ingest pollutants through the soil, unlike roe deer, which feed mainly on grass, leaves, berries and young shoots (Guckert et al. 2023; Warenik-Bany et al. 2016). This may explain why wild boar liver contains more frequent and higher PFAS concentrations than roe deer liver, both in our study and in others (Guckert et al. 2023). Guckert et al. (2023) summarised from samples from Denmark and Germany that PFAS concentrations were highest in carnivores, followed by omnivores including wild boar liver and herbivores including roe deer. However, despite lower concentrations, it is also possible to track temporal and spatial trends of PFAS using the liver of roe deer with significant decreases within ten years for PFOS (Falk et al. 2012).

The suitability as a bioindicator applies also to PCBs. Warenik-Bany et al. (2019) showed that the levels and types of pollutants as well as their relative abundance in deer and boar tissue reflected the surrounding environment and local pollutant emitters. They found a strong correlation between pollution in pine needles, leaves, grass, soil and tissue. Also, OC insecticides like DDT and HCH can be found in agricultural products such as cereals and vegetables showing contamination of soil samples (Łozowicka et al. 2016). Following the food chain, a broad spectrum of pesticides can be detected in wild boars but also in roe deer (Kaczyński et al. 2021). Accordingly, pollutants detected in the current study such as DDT and its metabolite DDE, HCH, permethrin and tebuconazole are known as common residues in wild boar and roe deer liver (Capcarova et al. 2019; Kaczyński et al. 2021). However, there is a lack of studies showing the extent to which the pesticide profile of the environment is reflected in game samples. For anticoagulant rodenticides, wild boar and roe deer are certainly not suitable as bioindicators, because they are used as biocides in urban areas or on farms instead of in agricultural fields. Red fox (*Vulpes vulpes*), a widespread generalist game species, can be a good focal species for anticoagulant exposure (Campbell et al. 2024; Geduhn et al. 2015).

## Conclusion

Analysis of 219 prominent persistent organic pollutants revealed that in total 24 pollutants consisting of PFAS, more persistent non-dioxin-like PCBs and pesticides (i.e. insecticides, fungicides and rodenticides) could be detected over all hunted game liver samples. The results support the possibility of pollution monitoring via hunted game species. Consequences for animal health cannot be conclusively assessed because of data gaps. An association between accumulation in the liver and animal health has only been established for anticoagulant rodenticides and the concentrations found in the present study are far lower than 200 µg kg^−1^.

In wild boar liver, concentrations of PFAS (sum of PFOS, PFOA and not quantified detected PFHxS) and the rodenticide brodifacoum exceeded tolerable weekly intake levels and acute reference doses for humans, respectively. Consumers who frequently eat wild game liver, such as hunters and their families, could have an elevated health risk due to the potentially delayed consequences of chronic exposure, but also because acute exposure effects seem possible depending on the number of portions consumed. As many chemical residues are typically accumulated in the liver and less likely in muscle tissue, the health risk resulting from the consumption of regular game meat is usually negligible (Kaczyński et al. 2021; Warenik-Bany et al. 2016). Future studies should focus on identifying to what extent pollutants are found in different environmental compartments, including soil (Röhler et al. 2021; Štrbac et al. 2024) and water (Ulrich et al. 2024) and all along the food chain from plants (Lesmeister et al. 2021) to animals (Rupp et al. 2023). Such studies would help to predict environmental pollution from game animals as well as the effects of measures undertaken in the name of animal health and consumer protection. This could, for example, quantify the benefit of soil remediation (Brunn et al. 2023).

## Supplementary Information

Below is the link to the electronic supplementary material.

ESM 1 (DOCX 112 KB)

## Data Availability

All data used are provided in the supporting information.
