# Supplementary material for: Differential prevalence of PFAS, PCBs and pesticides in liver of hunted game
Source: Environ Sci Pollut Res Int. 2026 May 15;33(17):8294–310. doi: 10.1007/s11356-026-37805-w (PMC13226317; doi:10.1007/s11356-026-37805-w)
Supplement: Supplementary file 1 — (DOCX 112 KB) [file 11356_2026_37805_MOESM1_ESM.docx]

**Supplementary**

**Differential prevalence of PFAS, PCBs and pesticides of hunted game – Supporting Table 1**

Alexandra Esther, Michelle Peter, Vera Ritz, Daniel Esther, Doreen Gabriel, Roman Trommler, Detlef Schenke, Klaus Polaczek, Kathrin Fisch, Christoph Müller

**Content**

**Table S1:** List of analysed pollutants

**S2 Anticoagulant rodenticides**

**Table S2_a:** Configuration of LC-MS/MS

**Table S2_b:** LC-MS/MS - MRM-conditions

**Table S2_c1:** FIRST ANALYSIS CAMPAIGN: Validation – Recovery

**Table S2_c2:** FIRST ANALYSIS CAMPAIGN: – Hunted liver samples

**Table S2_d1:** SECOND ANALYSIS CAMPAIGN: Validation – Recovery

**Table S2 _d2:** SECOND ANALYSIS CAMPAIGN: Validation – Hunted liver samples

**S3 Herbicide, herbicide safener, insecticide, fungicide, nematicide, microbiocide, plant growth regulator**

**Table S3_a:** Configuration of GC-MS/MS for the analysis of herbicide, herbicide safener, insecticide, fungicide, nematicide, microbiocide, plant growth regulator, PCB

**S4 PFAS**

**Table S4_a:** Configuration of LC-MS/MS for the analysis of PFAS

**Table S4_b:** LC-MS/MS - MRM-conditions for the analysis of PFAS

**Table S1** List of analysed pollutants including name, pollutant type, retention time (RT), MRM transitions, collision energy (CE) and LOQ (limit of quantification). Quantifier transitions are marked in bold. OC: organochlorine, PCB: polychlorinated biphenyl. Detected pollutants are highlighted in gray; in italics determined by LC-MS/MS, more detailed information on the MS/MS settings are provided in Supplement S2; n.a. not applicable; n.d. not determined; * The rodenticides were analysed in two sequences at different retention times and LOQs (Supporting Table S2).

| Name | Pollutant type | RT [min] | MRM transitions | CE  [eV] | LOQ [µg kg^-1^] |
| --- | --- | --- | --- | --- | --- |
| 2,4-D-ethyl ester | Herbicide | 7.49 | **247.9 🡪 185.0** 185.0 🡪 114.9  175.0 🡪 111.0 | 10  25 10 | n.d. |
| 2-Phenylphenol | Microbiocide | 6.25 | 169.1 🡪 91.0 **141.1 🡪 63.0** 115.1 🡪 65.0 | 35 45 25 | n.d. |
| 8-Hydroxyquinoline | Fungicide, Microbiocide | 5.38 | 145.0 🡪 63.0 117.0 🡪 63.0  **117.0 🡪 39.1** | 40  40  40 | n.d. |
| Acequinocyl | Insecticide | 16.77 | 342.9 🡪 188.8 **341.9 🡪 187.9**   187.9 🡪 131.0 | 20  15  20 | n.d. |
| Acetamiprid | Insecticide | 13.85 | 221.0 🡪 56.1 126.0 🡪 90.0  **126.0 🡪 72.9** | 15  5  20 | n.d. |
| Acibenzolar-S-methyl | Fungicide | 9.30 | 182.0 🡪 167.1  182.0 🡪 153.1 **182.0 🡪 135.0** | 10 10 15 | n.d. |
| Aclonifen | Herbicide | 12.39 | 264.1 🡪 194.2 **194.1 🡪 167.1** 194.1 🡪 139.1 | 15 20 25 | n.d. |
| Acrinathrin | Insecticide | 15.02 | **288.9 🡪 92.8** 207.8 🡪 152.0  181.0 🡪 127.0 | 10  35  30 | n.d. |
| Aldrin | Insecticide  (OC) | 9.94 | 262.9 🡪 192.9  262.9 🡪 190.9  254.9 🡪 220.0 | 35 35  20 | n.d. |
| Ametoctradin | Fungicide | 15.20 | 275.0 🡪 246.2 275.0 🡪 190.3  **246.0 🡪 188.2** | 0  15  25 | 20 |
| Amisulbrom | Fungicide | 16.14 | 227.9 🡪 147.0 **225.9 🡪 147.0** 214.0 🡪 160.0 | 15 15  20 | 4 |
| Azoxystrobin | Fungicide | 18.30 | 344.1 🡪 182.9 **344.1 🡪 171.9**  344.1 🡪 155.8 | 25  40  40 | 4 |
| Beflubutamid | Herbicide | 10.67 | 192.9 🡪 145.1  192.9 🡪 95.0 **176.1 🡪 79.1** | 15  35  25 | n.d. |
| Benalaxyl | Fungicide | 12.87 | 266.0 🡪 148.1 **233.9 🡪 146.0** 206.0 🡪 162.1 | 5  20  5 | n.d. |
| Bentazone | Herbicide | 10.11 | **225.0 🡪 181.9** 198.0 🡪 92.0 182.0 🡪 90.0 | 5  30  15 | n.d. |
| Benthiavalicarb-isopropyl | Fungicide | 14.57 | 222.0 🡪 125.9 **180.0 🡪 127.0** 180.0 🡪 83.0 | 40  20  30 | n.d. |
| Bifenazate | Insecticide | 13.94 | 184.1 🡪 91.1  **184.1 🡪 77.0**  168.1 🡪 140.1 | 40  40  10 | 20 |
| Bifenox | Herbicide | 14.21 | 340.9 🡪 309.9 **340.9 🡪 280.9**  189.1 🡪 126.0 | 10  15  20 | n.d. |
| Bifenthrin | Insecticide | 13.83 | **181.0 🡪 115.1** 166.0 🡪 139.1  166.0 🡪 115.1 | 45  35  35 | 4 |
| Boscalid | Fungicide | 16.50 | 140.0 🡪 112.0 **140.0 🡪 76.0**  111.9 🡪 76.0 | 10  25  15 | 1 |
| *Brodifacoum* | *Rodenticide* | **2.76*  *2.57* | ***521.0 🡪 78.8***  *521.0 🡪 135.0* | *-128*  *-46* | **2*  *1* |
| *Bromadiolone* | *Rodenticide* | **2.57*  *2.43* | ***527.0 🡪 250.0***  *525.0 🡪 181.0* | *-50*  *-46* | **2*  *1* |
| Bromoxynil | Herbicide | 7.41 | 276.8 🡪 88.0 **274.7 🡪 167.9** 274.7 🡪 88.0 | 30  15  30 | n.d. |
| Bromuconazole  (2 isomers) | Fungicide | 13.85  14.29 | 295.0 🡪 172.9 **293.0 🡪 172.9** 173.0 🡪 109.0 | 10  10  30 | n.d. |
| Bupirimate | Fungicide | 11.80 | **315.8 🡪 207.9** 208.0 🡪 68.9 193.0 🡪 109.0 | 10  30  15 | n.d. |
| Buprofezin | Insecticide | 11.74 | **304.9 🡪 175.0** 249.1 🡪 193.0 171.1 🡪 115.0 | 10  10  10 | n.d. |
| Captan | Fungicide | 10.73 | **263.8 🡪 79.0** 149.0 🡪 70.0 116.9 🡪 82.0 | 15  15  30 | n.d. |
| Carbetamide | Herbicide | 9.95 | 120.1 🡪 92.0  120.1 🡪 77.0  **119.1 🡪 64.1** | 10  15  25 | 20 |
| Carboxin | Fungicide | 11.75 | **234.9 🡪 143.0** 234.9 🡪 87.0 131.9 🡪 77.0 | 10  20  20 | n.d. |
| Carfentrazone-ethyl | Herbicide | 12.81 | **339.9 🡪 311.9** 329.9 🡪 309.9 311.9 🡪 150.8 | 10  10  20 | n.d. |
| *cis-*Chlordane | Insecticide  (OC) | 11.20 | 374.8 🡪 265.8 **372.8 🡪 265.8** 271.7 🡪 236.9 | 15 15  15 | 1 |
| *trans-*Chlordane | Insecticide  (OC) | 10.94 | 374.8 🡪 265.8 **372.8 🡪 265.8** 271.7 🡪 236.9 | 15  15  15 | 1 |
| Chloridazon (Pyrazon) | Herbicide | 13.04 | 221.0 🡪 220.2  **220.0 🡪 193.1** 220.0 🡪 166.0 | 5  20  25 | n.d. |
| *Chlorophacinone* | *Rodenticide* | **2.34*  *2.12* | ***373.0 🡪 201.0*** | *32* | **10*  *2* |
| Chlorothalonil | Fungicide | 8.54 | 265.9 🡪 230.9  **265.9 🡪 133.0** 265.9 🡪 109.0 | 20  45  45 | n.d. |
| Chlorotoluron | Herbicide | 9.70 | **212.1 🡪 166.0** 212.1 🡪 72.0  167.0 🡪 132.1 | 10  15  15 | n.d. |
| Chlorpropham | Herbicide, Plant growth regulator | 7.11 | **213.0 🡪 171.1** 171.0 🡪 127.1 153.0 🡪 90.0 | 5  5  25 | n.d. |
| Chlorpyrifos | Insecticide | 9.86 | **313.8 🡪 257.8** 196.9 🡪 107.0 196.9 🡪 98.0 | 15  40  30 | 1 |
| Chlorpyrifos-methyl | Insecticide | 9.14 | 287.9 🡪 92.9 **285.9 🡪 93.0** 124.9 🡪 47.0 | 20  25  15 | 1 |
| Clodinafop-propargyl | Plant growth regulator | 12.97 | 348.9 🡪 265.9  **348.9 🡪 237.8** 238.0 🡪 130.0 | 10  15  15 | n.d. |
| Clomazone | Herbicide | 7.98 | **205.1 🡪 107.1** 127.0 🡪 101.0 125.0 🡪 89.0 | 20  20  15 | n.d. |
| Cloquintocet-mexyl | Herbicide safener | 14.00 | 220.0 🡪 191.9 **163.0 🡪 128.0** 163.0 🡪 101.0 | 10  15  30 | n.d. |
| *Coumatetralyl* | *Rodenticide* | **2.18*  *1.92* | ***291.0 🡪 141.0***  *291.0 🡪 247.0* | *-38*  *-32* | **1*  *0.2* |
| Cyflufenamid | Fungicide | 11.88 | 188.1 🡪 88.0 **118.1 🡪 90.0** 118.1 🡪 89.0 | 35  10  25 | 4 |
| Cyfluthrin  (3 isomers) | Insecticide | 16.17  16.25  16.37 | 206.0 🡪 176.9 206.0 🡪 150.0  **162.9 🡪 127.0** | 25  40  5 | n.d. |
| Cyhalofop-butyl | Herbicide | 14.68 | **357.1 🡪 229.1** 256.2 🡪 120.1 229.2 🡪 109.1 | 15  10  15 | n.d. |
| Cyhalothrin (*gamma* and *lambda* isomer) | Insecticide | 14.79  14.60 | **208.0 🡪 181.0** 208.0 🡪 152.0  197.0 🡪 161.1 | 5  25  5 | 4 |
| Cypermethrin  (3 isomers) | Insecticide | 16.39  16.48  16.57 | 165.0 🡪 127.1  165.0 🡪 91.1 **162.9 🡪 127.0** | 0  10  0 | 4 |
| Cyproconazole | Fungicide | 11.99 | **222.0 🡪 124.9** 138.9 🡪 111.0 138.9 🡪 75.0 | 25  15  35 | n.d. |
| Cyprodinil | Fungicide | 10.39 | 225.2 🡪 224.3  **224.2 🡪 131.1** 210.0 🡪 93.0 | 10  15  20 | n.d. |
| Cyromazine | Insecticide | 7.97 | **165.9 🡪 109.0** 151.0 🡪 82.0 109.0 🡪 68.0 | 20  30  20 | n.d. |
| Dazomet | Fungicide | 7.76 | 161.9 🡪 89.0  **89.0 🡪 46.0**  88.9 🡪 74.0 | 25  5  15 | n.d. |
| *o,p‘-*DDD | Insecticide  (OC) (Breakdown) | 11.78 | 235.0 🡪 200.1 235.0 🡪 139.1 **199.1 🡪 164.1** | 10  45  20 | n.d. |
| *p,p‘-*DDD | Insecticide  (OC) (Breakdown) | 12.36 | 237.0 🡪 200.1 **199.1 🡪 164.1** 165.1 🡪 139.0 | 15  20  35 | n.d. |
| *o,p‘-*DDE | Insecticide  (OC) (Breakdown) | 10.98 | 317.8 🡪 248.0 248.0 🡪 176.2  **246.0 🡪 176.2** | 15  30  30 | n.d. |
| *p,p‘-*DDE | Insecticide  (OC)  (Breakdown) | 11.52 | 317.8 🡪 246.0 315.8 🡪 246.0  **246.1 🡪 176.2** | 15  15  30 | 1 |
| *o,p‘-*DDT | Insecticide  (OC) | 12.27 | 237.0 🡪 199.1 **235.0 🡪 199.1** 199.0 🡪 163.1 | 15  15  35 | n.d. |
| *p,p‘-*DDT | Insecticide  (OC) | 12.94 | 237.0 🡪 165.2 235.0 🡪 199.2  **235.0 🡪 165.2** | 20  15  20 | 2 |
| Deltamethrin | Insecticide | 18.02 | 252.9 🡪 174.0 **252.9 🡪 93.1** 251.0 🡪 172.0 | 0  15  0 | 4 |
| Desmedipham | Herbicide | 7.59 | 181.0 🡪 122.0 **181.0 🡪 109.0** 135.0 🡪 52.0 | 10  10  25 | n.d. |
| Diazinon | Insecticide | 8.29 | 276.0 🡪 137.1 **199.1 🡪 135.1** 179.1 🡪 137.1 | 25  10  20 | n.d. |
| Dicamba-methyl ester | Herbicide | 6.26 | 234.0 🡪 173.0 **205.0 🡪 149.0** 175.0 🡪 111.0 | 20  15  20 | n.d. |
| Diclofop-methyl | Herbicide | 13.26 | 339.9 🡪 252.9 **280.8 🡪 119.9** 253.0 🡪 162.1 | 10  10  15 | n.d. |
| Dieldrin | Insecticide  (OC) | 11.62 | **277.0 🡪 241.0**  262.9 🡪 193.0  262.9 🡪 191.0 | 5  35  35 | 2 |
| Diethofencarb | Fungicide | 9.76 | 225.0 🡪 96.0 **207.0 🡪 179.1** 207.0 🡪 151.0 | 30  5  15 | n.d. |
| *Difenacoum* | *Rodenticide* | **2.64*  *2.50* | ***443.0 🡪 135.0***  *443.0 🡪 93.0* | *-46*  *-80* | **1*  *0.2* |
| Difenoconazole  (2 isomers) | Fungicide | 17.72  17.78 | 324.8 🡪 266.8 **322.8 🡪 264.8** 264.9 🡪 202.0 | 15  15  20 | 4 |
| *Difethialone* | *Rodenticide* | **2.79*  *2.64* | ***539.0 🡪 80.8***  *539.0 🡪 151.0* | *-92*  *-46* | **1*  *1* |
| Diflubenzuron | Insecticide | 5.01 | 141.0 🡪 113.0  **141.0 🡪 63.0**  113.0 🡪 63.0 | 40  40  40 | 4 |
| Diflufenican | Herbicide | 13.29 | **393.9 🡪 265.9** 266.0 🡪 246.1 218.0 🡪 140.1 | 10  15  20 | n.d. |
| Dimethachlor | Herbicide | 8.99 | 209.9 🡪 134.1 **196.9 🡪 148.2** 134.1 🡪 79.1 | 10  10  20 | n.d. |
| Dimethenamide-P | Herbicide | 9.02 | 229.9 🡪 154.0  **229.9 🡪 111.0** 202.9 🡪 154.0 | 10  25  10 | n.d. |
| Dimethoate | Insecticide | 7.79 | 228.7 🡪 87.0  157.0 🡪 93.0  **157.0 🡪 63.0** | 5  10  25 | n.d. |
| Dimethomorph  (2 isomers) | Fungicide | 18.35  18.66 | 302.9 🡪 164.9 **300.9 🡪 165.0** 300.9 🡪 138.8 | 10  10  15 | 4 |
| Dimoxystrobin | Fungicide | 13.85 | 237.0 🡪 116.0 **205.0 🡪 116.0** 174.0 🡪 115.0 | 15  10  30 | n.d. |
| Diuron | Herbicide | 10.66 | 231.7 🡪 71.8 **186.9 🡪 124.0** 158.9 🡪 123.9 | 15  20  10 | n.d. |
| Dodemorph  (2 isomers) | Fungicide | 10.24  10.55 | **281.0 🡪 154.0** 238.1 🡪 55.1  154.0 🡪 112.1 | 10  20  10 | n.d. |
| Epoxiconazole | Fungicide | 13.52 | **192.0 🡪 138.1** 192.0 🡪 111.0  138.0 🡪 75.0 | 10  25  25 | 1 |
| Ethofenprox | Insecticide | 16.78 | 183.0 🡪 168.0  **163.0 🡪 135.1**  163.0 🡪 107.1 | 10  10  20 | n.d. |
| Ethofumesate | Herbicide | 9.61 | 285.9 🡪 207.1 **178.9 🡪 137.1** 178.9 🡪 105.1 | 5  0  15 | n.d. |
| Ethoprophos | Insecticide | 7.02 | 199.9 🡪 97.0 **157.9 🡪 97.0** 157.9 🡪 81.0 | 20  15  15 | n.d. |
| Etoxazole | Insecticide | 14.07 | 329.9 🡪 315.0 299.9 🡪 284.9  **299.9 🡪 269.9** | 20  10  20 | n.d. |
| Etridiazole | Fungicide | 5.85 | 211.1 🡪 183.0 211.1 🡪 140.0 **185.0 🡪 142.0** | 10  25  15 | n.d. |
| Famoxadone | Fungicide | 18.44 | 329.9 🡪 329.0  **329.9 🡪 223.9** 223.9 🡪 196.2 | 10  10  10 | n.d. |
| Fenamiphos | Insecticide | 11.31 | 302.9 🡪 287.9 302.9 🡪 153.9  **287.9 🡪 259.7** | 10  15  5 | n.d. |
| Fenazaquin | Insecticide | 14.19 | **160.0 🡪 145.2**  160.0 🡪 117.1  146.0 🡪 118.1 | 5  20  10 | 2 |
| Fenbuconazole | Fungicide | 16.21 | 197.9 🡪 129.0 197.9 🡪 102.0  **125.0 🡪 89.0** | 5  30  20 | n.d. |
| Fenhexamid | Fungicide | 12.97 | 301.0 🡪 97.0 179.0 🡪 115.0  **177.1 🡪 113.0** | 15  15  15 | 2 |
| Fenoxaprop-P-ethyl | Herbicide | 15.33 | 360.8 🡪 287.8 **287.8 🡪 118.8** 287.8 🡪 90.9 | 10  10  20 | n.d. |
| Fenoxycarb | Insecticide | 13.86 | 256.1 🡪 187.2 **186.2 🡪 109.0** 186.2 🡪 77.1 | 10  15  20 | n.d. |
| Fenpropidin | Fungicide | 9.45 | **273.0 🡪 98.0** 145.0 🡪 117.0  145.0 🡪 91.0 | 5  10  25 | 4 |
| Fenpropimorph | Fungicide | 9.81 | 128.1 🡪 110.1  128.1 🡪 86.1 **128.1 🡪 70.1** | 5  10  10 | 4 |
| Fenpyroximate | Insecticide | 7.85 | 212.0 🡪 185.0  **212.0 🡪 76.9** 198.1 🡪 114.0 | 40  40  35 | n.d. |
| Fenvalerate  (2 isomers) | Insecticide | 17.31  17.50 | 419.1 🡪 166.8 **167.0 🡪 125.1** 167.0 🡪 89.0 | 10  10  40 | 2 |
| Fipronil | Insecticide | 10.64 | **366.8 🡪 212.8** 350.8 🡪 254.8 254.9 🡪 228.0 | 25  15  15 | n.d. |
| Fipronil sulfide | Insecticide (Breakdown) | 10.50 | 420.0 🡪 350.9 **351.0 🡪 254.9** 254.9 🡪 156.9 | 10  20  35 | n.d. |
| Fipronil sulfone | Insecticide (Breakdown) | 11.71 | 384.8 🡪 256.8 **382.8 🡪 254.9** 254.9 🡪 227.9 | 20  20  15 | 2 |
| *Flocoumafen* | *Rodenticide* | **2.70*  *2.56* | ***541.0 🡪 382.0***  *541.0 🡪 161.0* | *-36*  *-48* | **1*  *0.4* |
| Fluazifop-p-butyl | Herbicide | 11.97 | 382.9 🡪 282.0 **281.9 🡪 238.0** 254.0 🡪 146.1 | 10  15  15 | 1 |
| Fludioxonil | Fungicide | 11.51 | 248.0 🡪 182.1  248.0 🡪 154.1 **248.0 🡪 127.1** | 10  20  30 | 1 |
| Flufenacet | Herbicide | 9.96 | 211.0 🡪 123.0  **211.0 🡪 96.0** 183.0 🡪 69.0 | 5  15  20 | n.d. |
| Flumetralin | Herbicide | 11.19 | 403.9 🡪 156.8 359.9 🡪 313.9  **157.0 🡪 109.0** | 15  15  25 | n.d. |
| Flumioxazin | Herbicide | 17.43 | 354.0 🡪 325.9 354.0 🡪 175.8  **287.0 🡪 258.7** | 5  15  15 | n.d. |
| Fluometuron | Herbicide | 6.98 | 232.0 🡪 72.0 **213.0 🡪 167.9** 187.0 🡪 109.0 | 15  10  20 | n.d. |
| Fluopyram | Fungicide | 10.57 | 395.9 🡪 223.1 **222.9 🡪 196.0** 222.9 🡪 187.1 | 5  10  10 | 1 |
| Fluorochloridone | Herbicide | 10.11 | 311.0 🡪 174.1  **311.0 🡪 102.9** 187.1 🡪 109.1 | 15  15  20 | n.d. |
| Flupyradifurone | Insecticide | 14.87 | 288.0 🡪 126.1 128.0 🡪 90.0  **126.0 🡪 73.0** | 15  10  25 | 20 |
| Fluquinconazole | Fungicide | 15.85 | 342.0 🡪 107.8 **340.0 🡪 298.0** 340.0 🡪 107.8 | 40  15  40 | n.d. |
| Fluroxypyr-meptyl | Herbicide | 13.29 | 237.0 🡪 209.0 **237.0 🡪 181.0** 208.9 🡪 178.9 | 5  15  20 | n.d. |
| Flurtamone | Herbicide | 14.43 | **332.7 🡪 120.0** 157.0 🡪 137.1  157.0 🡪 107.0 | 15  15  25 | n.d. |
| Flutolanil | Herbicide | 11.38 | 322.9 🡪 281.0 **280.9 🡪 173.0** 173.0 🡪 95.0 | 5  10  30 | n.d. |
| Flutriafol | Fungicide | 11.30 | **219.1 🡪 123.1** 219.1 🡪 95.0 164.1 🡪 109.1 | 15  35  20 | n.d. |
| *tau-*Fluvalinate (2 isomers) | Insecticide | 17.48  17.52 | 252.0 🡪 200.0 **250.0 🡪 200.1** 250.0 🡪 198.1 | 15  15  40 | 4 |
| Fluxapyroxad | Fungicide | 14.57 | **321.1 🡪 152.9** 222.0 🡪 152.9 222.0 🡪 125.9 | 35  15  40 | n.d. |
| Fosthiazate  (2 isomers) | Nematicide | 10.27  10.31 | **199.0 🡪 102.0** 195.0 🡪 60.0 165.9 🡪 106.0 | 5  20  10 | n.d. |
| Fuberidazole | Fungicide | 9.16 | 184.0 🡪 155.1 **156.0 🡪 103.1** 155.0 🡪 129.1 | 30  20  10 | n.d. |
| Haloxyfop-P-methyl | Herbicide | 10.93 | 375.1 🡪 316.0  **375.1 🡪 91.1** 288.0 🡪 180.0 | 10  35  25 | n.d. |
| *alpha-*HCH | Insecticide  (OC) | 7.64 | 218.9 🡪 183.0  **216.9 🡪 181.0** 180.9 🡪 145.0 | 5  5  15 | n.d. |
| *beta-*HCH | Insecticide  (OC) | 7.99 | 218.9 🡪 183.1 **216.9 🡪 181.1** 181.0 🡪 145.0 | 5  5  15 | 1 |
| *gamma-*HCH (Lindane) | Insecticide  (OC) | 8.08 | 218.9 🡪 183.1 **216.9 🡪 181.0** 181.0 🡪 145.0 | 5  5  15 | 1 |
| *delta-*HCH | Insecticide  (OC) | 8.51 | **217.0 🡪 181.1** 183.1 🡪 147.1  181.1 🡪 145.1 | 5  15  15 | n.d. |
| *epsilon-*HCH | Insecticide  (OC) | 8.69 | 254.0 🡪 180.9 218.9 🡪 182.9  **182.9 🡪 109.0** | 10  5  30 | n.d. |
| Heptachlor | Insecticide  (OC) | 9.34 | 273.7 🡪 238.9  273.7 🡪 236.9 **271.7 🡪 236.9** | 15  15  15 | 1 |
| Heptachlor endo-epoxide | Insecticide  (OC) (Breakdown) | 10.67 | 216.9 🡪 182.0  216.9 🡪 109.0 **183.0 🡪 119.0** | 20  45  30 | n.d. |
| Heptachlor exo-epoxide | Insecticide  (OC)  (Breakdown) | 10.61 | **354.8 🡪 264.9** 352.8 🡪 262.9  262.9 🡪 193.0 | 15  15  35 | n.d. |
| Hexachlorobenzene | Fungicide | 7.70 | **283.8 🡪 213.9** 281.8 🡪 211.9 248.9 🡪 179.0 | 30  30  30 | n.d. |
| Imazalil | Fungicide | 11.48 | 216.8 🡪 175.0 174.9 🡪 147.0  **172.9 🡪 109.0** | 5  15  30 | n.d. |
| Imidacloprid | Insecticide | 11.31 | 211.0 🡪 113.0 126.0 🡪 89.9  **126.0 🡪 73.0** | 15  5  25 | 20 |
| Indoxacarb | Insecticide | 18.02 | **264.0 🡪 175.8** 202.9 🡪 134.0  202.9 🡪 106.0 | 15  20  15 | n.d. |
| Ipconazole | Fungicide | 15.00 | 249.0 🡪 125.0 167.0 🡪 125.0  **125.0 🡪 89.0** | 15  5  20 | n.d. |
| Iprovalicarb  (2 isomers) | Fungicide | 11.61  11.78 | **158.0 🡪 98.0**  134.1 🡪 93.0  116.0 🡪 98.1 | 10  15  5 | n.d. |
| Isopyrazam | Fungicide | 15.30 | 359.0 🡪 159.0 **302.1 🡪 262.1** 159.0 🡪 139.0 | 40  15  10 | n.d. |
| Isoxaben | Herbicide | 15.17 | 165.0 🡪 150.0 165.0 🡪 107.0  **149.9 🡪 121.9** | 15  25  5 | n.d. |
| Kresoxim-methyl | Fungicide | 11.81 | 206.0 🡪 131.1 **206.0 🡪 116.0** 116.0 🡪 89.0 | 10  5  15 | n.d. |
| Lenacil | Herbicide | 12.95 | 233.9 🡪 153.1 153.1 🡪 110.1  **153.1 🡪 82.1** | 5  20  20 | 2 |
| Lufenuron | Insecticide | 5.58 | 251.6 🡪 157.8 **202.9 🡪 75.9** 173.9 🡪 109.9 | 15  40  30 | n.d. |
| Malathion | Insecticide | 9.73 | 172.9 🡪 117.0  **172.9 🡪 99.0** 157.8 🡪 125.0 | 15  10  5 | n.d. |
| MCPA-methyl ester | Herbicide | 6.51 | 214.1 🡪 155.1 214.1 🡪 141.1  **155.1 🡪 125.1** | 10  10  10 | n.d. |
| MCPB-methyl ester | Herbicide | 8.17 | 211.1 🡪 155.0  **142.1 🡪 107.1**  142.1 🡪 77.1 | 10  10  30 | n.d. |
| Mefenpyr-diethyl | Herbicide safener | 13.59 | **299.0 🡪 252.9** 253.0 🡪 190.0 253.0 🡪 189.0 | 10  20  30 | n.d. |
| Mepanipyrim | Fungicide | 11.16 | 222.2 🡪 158.1 221.2 🡪 220.2  **207.1 🡪 179.1** | 25  15  25 | n.d. |
| Metalaxyl | Fungicide | 9.33 | 234.0 🡪 146.1  **220.0 🡪 160.1** 206.1 🡪 162.1 | 20  10  5 | n.d. |
| Metamitron | Herbicide | 11.83 | **202.1 🡪 186.1** 202.1 🡪 104.1  173.1 🡪 132.1 | 5  15  10 | n.d. |
| Metazachlor | Herbicide | 10.45 | 209.0 🡪 133.2  **209.0 🡪 132.2** 209.0 🡪 117.1 | 10  15  35 | 1 |
| Metconazole | Fungicide | 14.22 | 153.1 🡪 125.0  153.1 🡪 70.0 **125.0 🡪 89.0** | 10  5  20 | n.d. |
| Methiocarb | Insecticide | 9.58 | 169.0 🡪 154.1 168.0 🡪 109.1  **153.0 🡪 91.1** | 10  15  20 | n.d. |
| Metobromuron | Herbicide | 8.79 | 258.0 🡪 61.0 **196.9 🡪 89.9** 169.9 🡪 142.9 | 10  25  20 | n.d. |
| *(S)-*Metolachlor | Herbicide | 9.89 | 238.0 🡪 162.2  **238.0 🡪 133.2** 162.1 🡪 133.2 | 10  30  15 | n.d. |
| Metrafenone | Fungicide | 15.24 | **394.8 🡪 364.8** 376.9 🡪 346.8 226.9 🡪 169.0 | 15  20  10 | 4 |
| Metribuzin | Herbicide | 9.00 | **198.0 🡪 82.0** 198.0 🡪 55.0  182.0 🡪 114.9 | 15  30  10 | n.d. |
| Myclobutanil | Fungicide | 11.68 | **179.0 🡪 125.1** 179.0 🡪 90.0  150.0 🡪 123.0 | 10  30  15 | 2 |
| Napropamide | Herbicide | 11.40 | **271.0 🡪 100.1**  271.0 🡪 72.1 128.0 🡪 100.1 | 15  15  10 | n.d. |
| Oryzalin | Herbicide | 15.51 | 316.8 🡪 274.9 **275.0 🡪 217.0** 258.0 🡪 193.9 | 5  5  5 | n.d. |
| Oxadiazon | Herbicide | 11.63 | **301.8 🡪 175.0** 257.8 🡪 112.0 174.9 🡪 112.0 | 15  30  15 | n.d. |
| Oxamyl | Insecticide, Nematicide | 6.30 | 162.0 🡪 114.9  145.0 🡪 71.9 **145.0 🡪 60.9** | 10  20  10 | n.d. |
| Oxychlordane | Insecticide  (OC) (Breakdown) | 10.53 | 386.7 🡪 262.7 236.9 🡪 142.9  **184.9 🡪 121.0** | 15  25  15 | 2 |
| Oxyfluorfen | Herbicide | 11.71 | 299.9 🡪 222.8 252.0 🡪 196.0  **252.0 🡪 146.0** | 15  20  30 | n.d. |
| Paclobutrazol | Plant growth regulator | 11.09 | 236.0 🡪 167.1 **167.1 🡪 132.1** 125.1 🡪 89.0 | 10  10  20 | n.d. |
| Parathion | Insecticide | 9.97 | **291.0 🡪 137.1** 291.0 🡪 109.0 139.0 🡪 81.0 | 5  15  15 | n.d. |
| Parathion-methyl | Insecticide, Nematicide | 9.14 | 262.9 🡪 109.0  262.9 🡪 79.0 **109.0 🡪 79.0** | 10  30  5 | n.d. |
| PCB 28 | PCB | 9.04 | **258.0 🡪 186.0** 256.0 🡪 186.0 186.0 🡪 151.0 | 25  25  25 | 1 |
| PCB 52 | PCB | 9.61 | 291.9 🡪 221.9 **289.9 🡪 219.9** 255.0 🡪 220.0 | 25  25  10 | 1 |
| PCB 101 | PCB | 11.12 | 325.9 🡪 255.9  325.9 🡪 253.9 **253.9 🡪 184.0** | 35  30  30 | 1 |
| PCB 138 | PCB | 13.12 | **361.9 🡪 289.9** 359.9 🡪 289.9 287.9 🡪 217.9 | 30  30  40 | 1 |
| PCB 153 | PCB | 12.62 | 361.9 🡪 289.9 **359.9 🡪 289.9** 287.9 🡪 217.9 | 25  25  40 | 1 |
| PCB 180 | PCB | 14.30 | 395.8 🡪 325.8 393.8 🡪 358.8  **393.8 🡪 323.8** | 30  15  30 | 1 |
| Penconazole | Fungicide | 10.54 | 250.0 🡪 194.1 **250.0 🡪 157.1** 159.0 🡪 123.0 | 15  25  20 | n.d. |
| Pendimethalin | Herbicide | 10.52 | **251.8 🡪 162.2** 251.8 🡪 146.1  161.9 🡪 147.0 | 10  20  10 | n.d. |
| Pentachloro-nitrobenzene | Fungicide, Nematicide | 8.20 | 294.8 🡪 236.8 248.8 🡪 213.8  **141.9 🡪 106.9** | 15  15  30 | 1 |
| *Perfluorooctanoic acid*  *(PFOA)* | *PFAS* | *3.8* | ***412.8 🡪 368.9***  *412.8 🡪 219.0*  *412.8 🡪 168.9* | *-16*  *-20*  *-22* | *8* |
| *Perfluoro-octaesulfonic acid (PFOS)* | *PFAS* | *2.3* | *498.8 🡪 129.9*  *498.8 🡪 98.8*  ***498.8 🡪 80.0*** | *-58*  *-58*  *-82* | *8* |
| *Perfluro-hexanesulfonic acid (PFHxS)* | *PFAS* | *2.7* | *398.7 🡪 118.9*  *398.7 🡪 98.9*  ***398.7 🡪 79.9*** | *-46*  *-48*  *-78* | *8* |
| Permethrin  (*cis* and *trans* isomer) | Insecticide | 15.51  15.63 | 165.0 🡪 127.0 **162.9 🡪 127.0** 162.9 🡪 91.0 | 0  0  10 | 4 |
| Phenmedipham | Herbicide | 7.08 | **167.0 🡪 135.0** 167.0 🡪 122.0  122.0 🡪 94.0 | 15  15  15 | n.d. |
| Phosmet | Insecticide | 13.90 | **161.0 🡪 134.0**  161.0 🡪 78.0  160.0 🡪 133.1 | 10  20  10 | n.d. |
| Phosmet-oxon | Insecticide (Breakdown) | 13.00 | **301.0 🡪 191.8** 172.9 🡪 104.0  160.0 🡪 133.0 | 10  15  15 | n.d. |
| Picloram-methyl ester | Herbicide | 9.55 | **198.0 🡪 163.1**  198.0 🡪 161.0  196.0 🡪 181.0 | 15  15  15 | n.d. |
| Picolinafen | Herbicide | 13.87 | 376.0 🡪 239.1  **376.0 🡪 238.1** 238.1 🡪 145.1 | 10  20  25 | 2 |
| Picoxystrobin | Fungicide | 11.29 | **334.9 🡪 172.9** 302.8 🡪 156.9 145.0 🡪 102.1 | 10  15  25 | n.d. |
| Pirimicarb | Insecticide | 8.73 | **238.0 🡪 166.2** 166.0 🡪 71.1 152.0 🡪 123.0 | 10  25  10 | 1 |
| Pirimiphos-methyl | Insecticide | 9.58 | 290.0 🡪 125.0 **232.9 🡪 151.0** 232.9 🡪 125.0 | 20  5  5 | n.d. |
| Prochloraz | Fungicide | 15.91 | 310.0 🡪 69.8 **266.0 🡪 69.9** 180.0 🡪 68.9 | 15  10  15 | n.d. |
| Propamocarb | Fungicide | 5.39 | 188.0 🡪 58.0 **143.0 🡪 99.1** 129.1 🡪 84.1 | 10  10  5 | n.d. |
| Propaquizafop | Herbicide | 19.92 | 298.8 🡪 254.8 **162.9 🡪 135.8** 162.9 🡪 99.9 | 25  10  20 | n.d. |
| Propiconazole  (2 isomers) | Fungicide | 12.89  13.00 | 258.8 🡪 172.9 172.9 🡪 109.0  **172.9 🡪 74.0** | 15  30  45 | 2 |
| Prosulfocarb | Herbicide | 9.37 | 251.0 🡪 218.3  **251.0 🡪 128.2** 251.0 🡪 100.1 | 10  5  5 | n.d. |
| Prothioconazole-desthio | Fungicide | 11.91 | **186.0 🡪 89.0** 186.0 🡪 70.0  125.0 🡪 99.0 | 10  10  20 | n.d. |
| Pymetrozine | Insecticide | 11.51 | 132.0 🡪 105.0  132.0 🡪 78.0  **113.0 🡪 98.0** | 10  20  5 | n.d. |
| Pyraclostrobin | Fungicide | 17.46 | **324.8 🡪 131.7** 164.0 🡪 132.1  110.8 🡪 75.0 | 15  10  15 | n.d. |
| Pyraflufen-ethyl | Herbicide | 13.03 | 412.0 🡪 349.0 349.0 🡪 307.0  **338.9 🡪 288.9** | 10  15  15 | n.d. |
| Pyridaben | Insecticide | 15.77 | **309.0 🡪 147.1** 147.2 🡪 132.2  147.2 🡪 117.1 | 15  10  20 | n.d. |
| Pyridalyl | Insecticide | 16.73 | 204.0 🡪 148.0 164.0 🡪 146.0  **146.0 🡪 126.0** | 25  15  10 | n.d. |
| Pyridate | Herbicide | 17.26 | **205.2 🡪 141.1** 205.2 🡪 114.0  205.2 🡪 102.0 | 25  35  30 | n.d. |
| Pyrimethanil | Fungicide | 8.24 | 198.0 🡪 183.1  198.0 🡪 158.1 **198.0 🡪 118.1** | 15  20  35 | 1 |
| Pyriproxyfen | Insecticide | 14.61 | **321.0 🡪 222.0** 321.0 🡪 153.0 136.1 🡪 96.0 | 10  25  15 | n.d. |
| Quinoclamine | Herbicide | 9.76 | 209.0 🡪 172.1 207.0 🡪 172.1  **172.0 🡪 89.0** | 10  20  20 | n.d. |
| Quinoxyfen | Fungicide | 12.85 | 306.8 🡪 237.0 271.9 🡪 237.1  **237.0 🡪 208.0** | 20  10  30 | 1 |
| Spirodiclofen | Insecticide | 15.56 | 312.1 🡪 259.0  **312.1 🡪 108.9** 157.0 🡪 73.0 | 10  15  25 | 2 |
| Spiromesifen | Insecticide | 13.71 | 272.0 🡪 209.2 **253.8 🡪 185.1** 231.0 🡪 157.1 | 10  15  15 | n.d. |
| Spiroxamine  (2 Isomers) | Fungicide | 9.08  9.53 | **198.0 🡪 126.1**  126.0 🡪 84.0  100.0 🡪 58.1 | 5  5  10 | n.d. |
| Tebuconazole | Fungicide | 13.22 | **250.0 🡪 125.0** 125.0 🡪 99.0  125.0 🡪 89.0 | 20  20  15 | 2 |
| Tebufenpyrad | Insecticide | 14.09 | 332.9 🡪 171.0 318.0 🡪 131.0  **275.9 🡪 171.1** | 15  15  10 | n.d. |
| Tefluthrin | Insecticide | 8.41 | 199.0 🡪 161.1 **197.0 🡪 161.1** 177.1 🡪 127.1 | 5  5  15 | n.d. |
| Terbuthylazine | Herbicide, Microbiocide | 8.12 | 228.9 🡪 138.0 214.0 🡪 71.0  **172.9 🡪 138.1** | 15  20  5 | 1 |
| Terbuthylazine-desethyl | Herbicide, Microbiocide | 7.36 | 186.2 🡪 104.0 186.2 🡪 83.1  **145.1 🡪 110.1** | 15  20  10 | 1 |
| Tetraconazole | Fungicide | 9.99 | 336.0 🡪 217.9  **336.0 🡪 203.8** 170.9 🡪 136.0 | 20  30  10 | 2 |
| Thiabendazole | Fungicide | 10.73 | **201.9 🡪 175.0** 201.0 🡪 130.0  173.9 🡪 65.0 | 15  30  30 | n.d. |
| Thiacloprid | Insecticide | 17.50 | 126.0 🡪 99.1  **126.0 🡪 90.1** 126.0 🡪 73.0 | 10  5  20 | n.d. |
| Tolclofos-methyl | Fungicide | 9.14 | **267.0 🡪 252.0** 267.0 🡪 93.0  267.0 🡪 63.0 | 15  30  45 | 2 |
| Tralkoxydim | Herbicide | 14.75 | 282.1 🡪 226.0 268.2 🡪 143.0  **226.0 🡪 143.0** | 10  40  25 | n.d. |
| Triadimenol | Fungicide | 10.73 | **129.9 🡪 102.0** 129.9 🡪 65.0  112.0 🡪 58.0 | 15  25  10 | n.d. |
| Triallate | Herbicide | 8.57 | **270.0 🡪 228.1** 268.0 🡪 226.1  268.0 🡪 184.1 | 10  10  20 | n.d. |
| Triclopyr-methyl ester | Herbicide | 7.51 | **209.9 🡪 145.9** 209.9 🡪 109.9  145.9 🡪 110.0 | 20  35  15 | n.d. |
| Trifloxystrobin | Fungicide | 12.94 | 186.0 🡪 145.1  **172.0 🡪 145.1**  172.0 🡪 95.0 | 15  15  30 | n.d. |
| Triflumizole | Fungicide | 10.81 | 345.0 🡪 302.0 239.1 🡪 66.9  **132.0 🡪 90.0** | 10  40  35 | n.d. |
| Trinexapac-ethyl | Herbicide | 9.50 | **224.0 🡪 151.0** 224.0 🡪 95.0 207.0 🡪 68.9 | 5  25  25 | n.d. |
| Triticonazole | Fungicide | 14.51 | 237.0 🡪 182.0 237.0 🡪 167.1  **234.8 🡪 182.1** | 10  25  10 | n.d. |
| *Warfarin* | *Rodenticide* | **2.27*  *2.07* | ***307.0 🡪 161.0***  *307.0 🡪 250.0* | *-26*  *-32* | **0.5*  *0.2* |
| Zoxamide | Fungicide | 13.47 | 259.9 🡪 189.0 **257.9 🡪 187.1** 189.0 🡪 161.1 | 10  10  15 | 4 |

**S2 Anticoagulant rodenticides**

**Table S2_a:** Configuration of LC-MS/MS

| **LIQUID CHROMATOGRAPHY** | **Agilent 1290 II Infinity** | | |
| --- | --- | --- | --- |
| Autosampler temperature | 10 °C | | |
| Injection volume | 5 µL | | |
| Analytical column | Agilent Zorbax Eclipse C18 (1.8 µm, 50 mm, 2.1 mm i.d.) | | |
| Column temperature | 40 °C | | |
| Mobile phase A | Water +1 mmol NH_4_F | | |
| Mobile phase B | Methanol /Acetonitrile (65/35) | | |
| Gradient program | Time (min) | A (%) | B (%) |
|  | 0.0 | 98 | 2 |
|  | 3.0 | 2 | 98 |
|  | 5.0 | 2 | 98 |
|  | 5.1 | 98 | 2 |
|  | 6.0 | 98 | 2 |
| Flow rate | 500 µL/min | | |
| **MASS SPECTROMETER** | **QTRAP 6500+ (SCIEX)** | | |
| Mode | negative ESI | | |
| Ion spray potential | -4500 V | | |
| Source temperature | 550 °C | | |
| Scan type | Multiple Reaction Monitoring (MRM) / Enhanced Product Ion (EPI) | | |
| Dwell time | 20 ms | | |
| Software | Analyst 1.7.1 | | |
| Quantification | relative peak area | | |

**Table S2_b:** LC-MS/MS - MRM-conditions of rodenticides, surrogates (Surr) and internal standards (IS); precursor (Q1), product ions (Q3) and mass range for EPI in *m/z* and declustering potential (DP), entrance potential (EP), collision energy (CE) and cell exit potential (CXP) in all in [V]; Quantifier transitions are marked in **bold**. EPI – enhanced product ion for complete MS/MS spectra.

| **Q1 Mass** | **Q3 Mass** | **Analyte** | **DP** | **EP** | **CE** | **CXP** | **IS Assignment** |
| --- | --- | --- | --- | --- | --- | --- | --- |
| 377.0 | 201.0 | Chlorophacinone-*d*_4_ (IS) | -120 | -10 | -32 | -15 |  |
| 312.0 | 161.0 | Warfarin-*d*_5_ (IS) | -95 | -10 | -28 | -9 |  |
| 352.0 | 265.0 | **Acenocoumarol (Surr)** | -70 | -10 | -40 | -13 | Warfarin-*d*_5_ |
| 341.0 | 160.0 | **Coumachlor (Surr)** | -60 | -10 | -30 | -21 | Warfarin-*d*_5_ |
| 343.0 | 167.0 | **Diphacinone-*d*_4_ (Surr)** | -115 | -10 | -32 | -15 | Chlorophacinone-*d*_4_ |
| 279.0 | 250.0 | **Phenprocoumon (Surr)** | -55 | -10 | -32 | -17 | Warfarin-*d*_5_ |
| **521.0** | **78.8** | **Brodifacoum** | **-20** | **-10** | **-128** | **-11** | Warfarin-*d*_5_ |
| 521.0 | 135.0 |  | -180 | -10 | -46 | -17 | Warfarin-*d*_5_ |
| **527.0** | **250.0** | **Bromadiolone** | **-30** | **-10** | **-50** | **-19** | Warfarin-*d*_5_ |
| 525.0 | 181.0 |  | -85 | -10 | -46 | -9 | Warfarin-*d*_5_ |
| **373.0** | **201.0** | **Chlorophacinone** | **-75** | **-10** | **-30** | **-13** | Chlorophacinone-*d*_4_ |
| **291.0** | **141.0** | **Coumatetralyl** | **-125** | **-10** | **-38** | **-13** | Warfarin-*d*_5_ |
| 291.0 | 247.0 |  | -125 | -10 | -32 | -21 | Warfarin-*d*_5_ |
| **443.0** | **135.0** | **Difenacoum** | **-55** | **-10** | **-46** | **-15** | Warfarin-*d*_5_ |
| 443.0 | 93.0 |  | -55 | -10 | -80 | -15 | Warfarin-*d*_5_ |
| **539.0** | **80.8** | **Difethialone** | **-20** | **-10** | **-92** | **-13** | Warfarin-*d*_5_ |
| 539.0 | 151.0 |  | -20 | -10 | -46 | -13 | Warfarin-*d*_5_ |
| **541.0** | **382.0** | **Flocoumafen** | **-65** | **-10** | **-36** | **-29** | Warfarin-*d*_5_ |
| 541.0 | 161.0 |  | -65 | -10 | -48 | -17 | Warfarin-*d*_5_ |
| **307.0** | **161.0** | **Warfarin** | **-45** | **-10** | **-26** | **-19** | Warfarin-*d*_5_ |
| 307.0 | 250.0 |  | -90 | -10 | -32 | -19 | Warfarin-*d*_5_ |
| **EPI- mass range** | | | **DP** | **EP** | **CE** |  |  |
| [m/z] |  |  | [V] | | | |  |
| 50 - 450 |  |  | -50 | -10 | -30(±15) |  |  |

**Table S2_c1:** FIRST ANALYSIS CAMPAIGN: Validation – Recovery with pig liver (n = 5), Limit of quantification (LOQ) and recovery levels (Rec) ± relative standard deviations (RSD). No findings in control samples (n = 2).

| **Analyte** | **LOQ** | **5**  [µg kg^-1^] | | **10**  [µg kg^-1^] | | **1000**  [µg kg^-1^] | |
| --- | --- | --- | --- | --- | --- | --- | --- |
|  | **[µg kg^-1^]** | **Rec** | **RSD** | **Rec** | **RSD** | **Rec** | **RSD** |
|  |  | **[%]** | **[%]** | **[%]** | **[%]** | **[%]** | **[%]** |
| Brodifacoum | 2.0 | 60 | 9 | 65 | 7 | 65 | 3 |
| Bromadiolone | 2.0 | 76 | 13 | 81 | 7 | 83 | 3 |
| Chlorophacinone | 10.0 | 37 | 18 | 63 | 8 | 81 | 3 |
| Coumatetralyl | 1.0 | 94 | 7 | 95 | 8 | 99 | 4 |
| Difenacoum | 1.0 | 85 | 6 | 85 | 5 | 86 | 3 |
| Difethialone | 1.0 | 45 | 6 | 52 | 12 | 53 | 1 |
| Flocoumafen | 1.0 | 80 | 7 | 82 | 6 | 80 | 1 |
| Warfarin | 0.5 | 101 | 7 | 102 | 5 | 107 | 4 |

**Table S2_c2:** FIRST ANALYSIS CAMPAIGN: Validation – Hunted liver samples batch (Recovery of Surrogates, 0.1 µg/sample) in quality control samples (pig liver, n = 17) and in hunted liver samples (n = 88), recovery levels (Rec) ± relative standard deviations (RSD).

| **Surrogate** | **Quality control** | | **Hunted liver samples** | |
| --- | --- | --- | --- | --- |
|  | **Rec** | **RSD** | **Rec** | **RSD** |
|  | **[%]** | **[%]** | **[%]** | **[%]** |
| Acenocoumarol | 99 | 10 | 98 | 13 |
| Coumachlor | 100 | 9 | 97 | 12 |
| Diphacinone-*d*_4_ | 81 | 8 | 91 | 15 |
| Phenprocoumon | 84 | 9 | 75 | 14 |

**Table S2_d1:** SECOND ANALYSIS CAMPAIGN: Validation – Recovery with calf liver (n = 10), LOQ and recovery levels (Rec) ± relative standard deviations (RSD). No findings in control samples (n = 2).

| **Analyte** | **LOQ** | **Quality control**  **100 [**µg kg^-1^] | | |
| --- | --- | --- | --- | --- |
|  |  | **Rec** | **RSD** |  |
|  | **[**µg kg^-1^**]** | **[%]** | **[%]** |  |
| Brodifacoum | 1.0 | 93 | 26 |  |
| Bromadiolone | 1.0 | 90 | 26 |  |
| Chlorophacinone | 2.0 | 109 | 8 |  |
| Coumatetralyl | 0.2 | 112 | 12 |  |
| Difenacoum | 0.2 | 90 | 35 |  |
| Difethialone | 1.0 | 80 | 34 |  |
| Flocoumafen | 0.4 | 87 | 41 |  |
| Warfarin | 0.2 | 128 | 7 |  |

**Table S2_d2:** SECOND ANALYSIS CAMPAIGN: Validation – Hunted liver samples batch (Recovery of Surrogates, 0.1 µg/sample) in quality control samples (calf liver, n = 10) and in hunted liver samples (n = 97), recovery levels (Rec) ± relative standard deviations (RSD).

| **Surrogate** | **Quality control** | | **Hunted liver samples** | |
| --- | --- | --- | --- | --- |
|  | **Rec** | **RSD** | **Rec** | **RSD** |
|  | **[%]** | **[%]** | **[%]** | **[%]** |
| Acenocoumarol | 127 | 12 | 114 | 13 |
| Coumachlor | 128 | 8 | 115 | 12 |
| Diphacinone-*d_4_* | 128 | 29 | 111 | 28 |
| Phenprocoumon | 108 | 13 | 93 | 17 |

**Table S3_a:** Configuration of GC-MS/MS for the analysis of herbicide, herbicide safener, insecticide, fungicide, nematicide, microbiocide, plant growth regulator, PCB

| **GAS CHROMATOGRAPHY** | **Agilent 7890** | | | |
| --- | --- | --- | --- | --- |
| Autosampler temperature | room temperature | | | |
| Injection volume | 1 µL | | | |
| Analytical column | 2 Agilent HP-5ms  ultra inert columns (15 m × 0.25 mm × 0.25 μm) | | | |
| Split ratio | splitless | | | |
| Inlet gradient program | Time (min) | Rate  (°C min^-1^) | Value  (°C) | Hold time  (min) |
|  | 0 | - | 60 | 0.2 |
|  | 0.2 | 900 | 280 | 0 |
| Oven gradient program | Time (min) | Rate  (°C min^-1^) | Value  (°C) | Hold time  (min) |
|  | 0 | - | 60 | 1 |
|  | 1 | 40 | 170 | 0 |
|  | 3.75 | 10 | 310 | 3 |
| Run time | 20.75 min | | | |
| Post run time | 5 min | | | |
| GC cycle time | 31 min | | | |
| Carrier gas | Helium 5.0 (Air Liquide) | | | |
| Carrier gas flow rate | Column 1: 0.9 mL min^-1^ Column 2: 1.1 mL min^-1^ | | | |
| Back flush flow rate | Column 1: -4.0 mL min^-1^ Column 2: 4.4 mL min^-1^ | | | |
| Vent flow | 100 mL min^-1^ | | | |
| **MASS SPEKTROMETER** | **7010B (AGILENT)** | | | |
| Mode | EI, positive | | | |
| Ion source voltage | 70 eV | | | |
| Source temperature | 230 °C | | | |
| Transfer line temperature | 280 °C | | | |
| Quadrupole temperature | 150° Q1 and Q2 | | | |
| Collision gas | Argon 4.5 (Air Liquide) 0.9 mL min^-1^ | | | |
| Quench gas | Helium 5.0 (Air Liquide) 2.25 mL min^-1^ | | | |
| Solvent delay | 4 min | | | |
| Scan type | multiple reaction monitoring (MRM) | | | |
| Software | Agilent Mass Hunter B.08.00 | | | |
| Quantification | relative peak area | | | |

**S4 PFAS**

**Table S4_a:** Configuration of LC-MS/MS for the analysis of PFAS

| **LIQUID CHROMATOGRAPHY** | **Agilent 1200** | | |
| --- | --- | --- | --- |
| Autosampler temperature | room temperature | | |
| Injection volume | 10 µL | | |
| Analytical column | Restek Raptor Polar X (50 × 2.1 mm, 2.5 µm) | | |
| Column temperature | 40 °C | | |
| Mobile phase A | 0.5% *(v/v)* formic acid in water | | |
| Mobile phase B | 0.5% *(v/v)* formic acid in acetonitril | | |
| Gradient program | isocratic | 25% A | 75% B |
| Flow rate | 0.5 mL min^-1^ | | |
| **MASS SPEKTROMETER** | **API3200 (SCIEX)** | | |
| Mode | negative ESI | | |
| Ion spray potential | -4500 V | | |
| Source temperature | 500 °C | | |
| Scan type | multiple reaction monitoring (MRM) | | |
| Dwell time | Quantifier 100 ms Qualifier 20 ms | | |
| Software | Analyst 1.6.1 | | |
| Quantification | relative peak area | | |

**Table S4_b:** LC-MS/MS - MRM-conditions of the analytes and internal standard for the analysis of PFAS; precursor (Q1), product ions (Q3) in *m/z;* declustering potential (DP), entrance potential (EP), collision energy (CE) and cell exit potential (CXP) in V; Quantifier transitions are marked in **bold**.

| **Q1 Mass** | **Q3 Mass** | **Analyte** | **DP** | **EP** | **CE** | **CXP** | **IS Assignment** |
| --- | --- | --- | --- | --- | --- | --- | --- |
| 265.0 | 97.0 | Sodium dodecyl sulfate (SDS, IS) | -55 | -7 | -40 | 0 |  |
| 265.0 | 96.3 |  | -55 | -7 | -78 | -58 |  |
| **265.0** | **80.0** |  | **-55** | **-7** | **-76** | **0** |  |
| **412.8** | **368.9** | Perfluorooctanoic acid  (PFOA) | **-30** | **-4.5** | **-16** | **-4** | SDS |
| 412.8 | 219.0 |  | -30 | -4.5 | -20 | -4 |  |
| 412.8 | 168.9 |  | -30 | -4.5 | -22 | 0 |  |
| 498.8 | 129.9 | Perfluorooctaesulfonic acid (PFOS) | -100 | -9 | -58 | 0 | SDS |
| 498.8 | 98.8 |  | -100 | -9 | -58 | -2 |  |
| **498.8** | **80.0** |  | **-100** | **-9** | **-82** | **-2** |  |
| 398.7 | 118.9 | Perflurohexanesulfonic acid (PFHxS) | -75 | -5.5 | -46 | 0 | SDS |
| 398.7 | 98.9 |  | -75 | -5.5 | -48 | 0 |  |
| **398.7** | **79.9** |  | **-75** | **-5.5** | **-78** | **0** |  |
